# B-cell intrinsic regulation of antibody mediated immunity by histone H2A deubiquitinase BAP1

**DOI:** 10.3389/fimmu.2024.1353138

**Published:** 2024-03-11

**Authors:** Yue Liang, HanChen Wang, Noé Seija, Yun Hsiao Lin, Lin Tze Tung, Javier M. Di Noia, David Langlais, Anastasia Nijnik

**Affiliations:** ^1^ Department of Physiology, McGill University, Montreal, QC, Canada; ^2^ McGill University Research Centre on Complex Traits, McGill University, Montreal, QC, Canada; ^3^ McGill Genome Centre, Montreal, QC, Canada; ^4^ Department of Human Genetics, McGill University, Montreal, QC, Canada; ^5^ Institut de Recherches Cliniques de Montréal, Montreal, QC, Canada; ^6^ Molecular Biology Programs, Université de Montréal, Montreal, QC, Canada; ^7^ Department of Medicine, Université de Montréal, Montreal, QC, Canada; ^8^ Department of Microbiology and Immunology, McGill University, Montreal, QC, Canada

**Keywords:** B cell, humoral immune response, epigenetic regulation and gene expression, mouse model, germinal center (GC) B cells, class switch recombination, deubiquinating enzymes

## Abstract

**Introduction:**

BAP1 is a deubiquitinase (DUB) of the Ubiquitin C-terminal Hydrolase (UCH) family that regulates gene expression and other cellular processes, through its direct catalytic activity on the repressive epigenetic mark histone H2AK119ub, as well as on several other substrates. BAP1 is also a highly important tumor suppressor, expressed and functional across many cell types and tissues. In recent work, we demonstrated a cell intrinsic role of BAP1 in the B cell lineage development in murine bone marrow, however the role of BAP1 in the regulation of B cell mediated humoral immune response has not been previously explored.

**Methods and results:**

In the current study, we demonstrate that a B-cell intrinsic loss of BAP1 in activated B cells in the *Bap1* ^fl/fl^ *Cγ1*-cre murine model results in a severe defect in antibody production, with altered dynamics of germinal centre B cell, memory B cell, and plasma cell numbers. At the cellular and molecular level, BAP1 was dispensable for B cell immunoglobulin class switching but resulted in an impaired proliferation of activated B cells, with genome-wide dysregulation in histone H2AK119ub levels and gene expression.

**Conclusion and discussion:**

In summary, our study establishes the B-cell intrinsic role of BAP1 in antibody mediated immune response and indicates its central role in the regulation of the genome-wide landscapes of histone H2AK119ub and downstream transcriptional programs of B cell activation and humoral immunity.

## Introduction

B lymphocytes are the central mediators of humoral immunity in response to infections and vaccinations (1). During humoral immune response naïve B cells make a multitude of cell fate decisions and differentiate into germinal center (GC) B cells, memory B cells, and plasma cells. Such differentiation pathways are tightly controlled by a complex network of transcriptional regulators, as for example PAX5 acts to maintain the B cell lineage identity, BCL6 drives GC transcriptional programs (2), and BLIMP1 and XBP1 promote plasma cell differentiation (3). Disruption of such transcriptional programs can result in life-threatening immunodeficiencies or carcinogenesis (1, 4).

Epigenetic regulators cooperate with transcription factors in the control of cellular gene expression. Polycomb repressive complexes (PRCs) are the central regulators of cell identity and differentiation (5, 6), and include the histone H3K27me3 methyltransferase PRC2 and the histone H2AK119ub E3 ubiquitin ligase PRC1 (7). While PRC2 is required for the normal induction of GC reaction and humoral immunity (8–11), and is widely investigated for its roles in lymphomagenesis and as a lymphoma drug target (4, 12, 13), the roles of PRC1 in humoral immunity are less well explored (14). The B-cell restricted loss of the PRC1 subunit BMI1 (also known as PCGF4) modulated GC reaction and enhanced humoral immunity in the context of chronic viral infections (15), while the loss of PRC1-binding transcription factor YY1 disrupted the progression of the GC reaction and impaired humoral immunity (16–18).

H2AK119ub is a highly abundant repressive epigenetic mark, estimated to occur on ~10% of nucleosomes in a typical mammalian cell (19). Thus, multiple chromatin-associated deubiquitinases are known to antagonize PRC1 by removing H2AK119ub from chromatin (H2A-DUBs) (20, 21). In previous work, we and others explored the roles of the DUB MYSM1 in B cell development (22, 23), humoral immune response (24), and B cell carcinogenesis (25), while others reported on the role of USP16 in hematopoiesis and the regulation of transcriptional programs in naïve B cells (26, 27). It is however important to stress that such DUBs are multifunctional proteins with many known substrates, and their specific roles in the regulation of the genome-wide H2AK119ub epigenetic landscapes or specific functions in the cell-fate decisions of the GC reaction and humoral immunity are poorly understood (20, 21).

BAP1 is a member of the ubiquitin C-terminal hydrolase (UCH) family of DUBs and is widely recognized as one of the major DUBs for H2AK119ub (28). At chromatin BAP1 acts in complex with ASXLs, HCF1 and OGT (29–32), and also interacts with transcription factors FOXK1/2 (33, 34), YY1 (35), KLF5 (36), and PGC1α (37). However, beyond the role of BAP1 as an epigenetic regulator of gene expression, it is known to have other substrates and functions both in the nucleus and the cytosol (28), as for example promoting apoptosis through its DUB catalytic activity on IP3R3 (38) and in DNA damage repair through the homologous recombination (HR) pathway (39).

BAP1 is an important tumor suppressor, mutated in sporadic uveal melanomas, mesotheliomas, renal cell carcinomas, hepatocellular carcinomas, and to a lesser extent other cancers (28, 40–42), while BAP1-interacting ASXL proteins are commonly mutated in myeloproliferative disorders (43, 44). Furthermore, rare *BAP1* germline mutations result in the so called ‘BAP1 cancer syndrome’, with a high incidence of uveal melanoma, mesothelioma, and other malignancies (28, 45, 46). In mouse models, BAP1 is known to be essential for normal progression of embryonic development, and its constitutive loss results in lethality at E9.5 of gestation (47). Interestingly, an inducible loss of BAP in the *Bap1* ^fl/fl^ Cre^ERT2^ mouse model resulted in hematologic and immune pathology, including myelodysplastic syndrome (47), impaired thymocyte development (48), and defective peripheral T cell expansion (48), with global increases in the H2AK119ub levels in the affected cells.

In recent work, we analyzed the role of BAP1 in B cells using the *Bap1* ^fl/fl^ mb1-*Cre* mouse model, with a selective loss of BAP1 throughout the B cell lineage (49). We demonstrated the cell-intrinsic requirement for BAP1 in the normal progression of B cell development, and established its role as a DUB for histone H2AK119ub in the regulation of the transcriptional programs of cell proliferation in pre-B cells (49).While BAP1 remains highly expressed throughout the B cell lineage (50), its role in the regulation of B cell activation and humoral immune response remains unknown and is the focus of our current work.

## Materials and methods

### Mice

The *Bap1* ^tm1c(EUCOMM)Hmgu^ mouse strain carries a conditional (floxed) allele of the *Bap1* gene; it was generated by the Wellcome Trust Sanger Institute Mouse Genetics Project and Infrafrontier/EMMA (www.infrafrontier.eu) (51–53), and described in our recent work (49). The strain was bred to the transgenic line expressing Cre recombinase under the control of the B cell lineage specific promoter mb1-*Cre* (from Prof. Michael Reth, MPI für Immunbiologie und Epigenetik, Germany) (54), and to the B6.129P2(Cg)-*Ighg1^tm1(cre)Cgn^*/J mouse strain (Jackson Labs: 010611, Cγ1-cre) expresses Cre recombinase from the endogenous immunoglobulin heavy constant gamma 1 locus (*Ighg1*) (55). As we previously described (49), the loxP sites flank exons 6-12 (ENSMUSE00000121807-00000121801) of the main ENSMUST00000022458.10 *Bap1* transcript, and the *Bap1* ^Δ^ allele is predicted to disrupt BAP1 protein coding sequence from amino acid 126 (out of 728), precluding full expression of the N-terminal UCH catalytic domain and all the downstream domains of the protein. All lines were on the C57BL/6 genetic background. Both male and female animals were used in experiments, sex-matched between test and control groups. Experiments were in accordance with the guidelines of the Canadian Council on Animal Care and protocol MCGL-2011-6029 approved by the McGill Animal Care Committee. Genotyping was performed in house using the DreamTaq DNA Polymerase (Thermo Fisher Scientific) and primers from IDT Technologies.

### Cell culture

Mouse B cell line CH12F3, which is a widely used model for the study of B cell activation and class switching (56), was maintained at 0.5-2 x10^6^ cells/mL in RPMI-1640 (Thermo Fisher Scientific) with 10% Fetal Calf Serum (FCS, Thermo Fisher Scientific), 2mM L-Glutamine, 100μg/mL streptomycin, 100U/mL penicillin (Thermo Fisher Scientific), and 5% NCTC (Sigma-Aldrich).

### CRISPR-Cas9 gene targeting

*Bap1* ^Δ/Δ^ CH12F3 cells were generated through CRISPR-Cas9 gene editing according to our previously described protocols (49). gRNAs were designed with http://crispr.mit.edu online tools (57): gRNA_Bap1_Exon4_Chr14:32,066,155: *gcaaatggatcgaagagcgc*, gRNA_Bap1_Exon5_Chr14: 32,066,654: *ggcgtgagtggcacaagagt*, and cloned into the pSpCas9(BB)-2A-GFP (PX458) plasmid (Addgene, #48138). Sequence-verified plasmids were introduced into CH12F3 cells through nucleofection using the Amaxa Cell Line Nucleofector Kit V (Lonza) according to the manufacturer’s protocol. Single GFP^+^ IgA^-^ cells were sorted on day-2, and expanded for further 14 days to generate single cell clones. Loss of BAP1 protein was validated with Western blotting and the following antibodies: anti-BAP1 (D7W7O, Cell Signaling Technology) and anti-β-Actin (D6A8, Cell Signaling Technology).

### CH12F3 cell assays

AlamarBlue fluorogenic assay (ThermoFisher Scientific) was performed according to the manufacturer’s protocols, as we previously described (49). Cells were seeded into 96-well plates at 10^5/^mL, rested overnight, and the AlamarBlue reagent added at 10% (v/v). Fluorescence intensity was recorded at the 4-hour timepoint on the EnSpire Plate reader (Perkin Elmer), at the 560nm excitation and 590nm emission wavelengths. For the CFSE-dilution assay, 5 × 10^6^ cells were incubated in 2 μM CellTrace™ CFSE (ThermoFisher Scientific) in 1mL of PBS for 10 min at 37°C. The cells were washed in PBS with 5% FBS, followed by a second wash in PBS. The cells were re-suspended in complete media at 2 × 10^5^/mL and maintained in culture overnight at 37°C 5% CO_2_. The cells were counterstained with Fixable Viability Dye eFluo780 (ThermoFisher Scientific), and the data were acquired on the BD Fortessa and analyzed with FlowJo (Tree Star, BD Biosciences) software. In some assays the CH12F3 cells were stimulated with TGF-β (1ng/ml, R&D Systems), IL-4 (10 ng/ml, Peprotech), and anti–CD40 (1μg/ml, BD Biosciences).

### Primary B cell cultures

The protocols were as described in our recent work (58). Briefly, naïve primary mouse B cells were purified from splenocytes using EasySep™ Mouse B Cell Isolation Kit (19854, Stem Cell Technologies) and were cultured at 37°C with 5% CO_2_ in iGB media: RPMI-1640 (Wisent), supplemented with 10% FBS (Wisent), 1% penicillin/streptomycin (Wisent), 0.1 mM 2-mercaptoethanol (BioShop), 10 mM HEPES, and 1 mM sodium pyruvate. Mouse primary B cells were labeled with 2.5μM CellTrace™ Violet (C34557, Invitrogen) in PBS for 20 minutes at 37°C before quenching as recommended by the supplier. For switching to IgA, B cells were stimulated with mouse recombinant IL-21 (20ng/mL, Peprotech), TGF-β1 (5ng/mL), retinoic acid (1μM, Sigma), F(ab’)2 goat anti-mouse IgM (5μg/mL, Jackson ImmunoResearch) and anti-CD40 mAb FGK45 (5μg/mL, prepared in-house from hybridoma), as previously described (59). To measure class switching to IgA, cells were treated with mouse FcR blocking reagent (130-092-575, Miltenyi Biotec) then stained with anti IgA-PE (1040-09, Southern Biotech). Dead cells were excluded using eBioscience™ Fixable Viability Dye eFluor™ 780 (65-0865-14, Invitrogen). Induced GC B cells (iGBs) were generated on 40LB feeder cells (a gift from Dr. Daisuke Kitamura, Tokyo University of Science, Tokyo, Japan) (60). One day prior to B cell plating, 40LB cells were treated with 10μg/mL mitomycin C (SKU M-1108, AG Scientific) at 0.5 × 10^6^ cells per mL in 10cm dishes with DMEM media supplemented with 10% FBS (Wisent) and 1% penicillin/streptomycin (Wisent). After treatment, the cells were washed 6 times with 10 mL of PBS and plated at 1.3×10^5^ cells per well in 0.5 mL (24-well plate). Subsequently, purified naive B cells were plated onto the 40LB feeders at 2x10^5^ cells in 1mL of iGB media, supplemented with 1 ng/mL IL-4 (214-14, Peprotech). On days 3, 4 and 5, the cells were either harvested for downstream analyses in PBS with 0.5% BSA and 2 mM EDTA or fed with 1 mL of fresh iGB media with 1 ng/mL IL-4. Class switching to IgG1 was analyzed by flow cytometry, treating with mouse FcR blocking reagent and staining the cells with anti-IgG1 PE (550083, A85-1, BD Pharmingen) and anti-IgM BV421 (562595, R6-60.2, BD Pharmingen). For the analyses of cell division, iGBs were stained with 2.5μM CellTrace™ Violet on the day of plating, according to the manufacturer’s protocol. Cell numbers for the iGBs growth curves were calculated using Countess 3 Automated Cell Counter.

### Mouse immunization

For the phycoerythrin (PE) immunization the mice were injected subcutaneously with immunogen emulsion comprising 100 μl CFA (Thermo Fisher Scientific), 15 μg of R-PE (ProZyme, Cedarlane), and 100 μl PBS, vortexed for 45 min prior to the injection. For the immunization with sheep red blood cells (SRBCs), the mice were injected intravenously with 10^9^ SRBCs (Innovative Research IC10-0210, Cedarlane) in 300uls of PBS. Immunization protocols were previously described (24, 61).

### Analyses of antibody titers

ELISA analyses of total immunoglobulin levels in the serum of naive mice (or in the supernatants from B cell cultures) used rat anti-mouse capture antibodies IgM II/41, IgG1 A85-3, and IgG3 R2-38 (BD Biosciences, 1 μg/ml) followed by detection with goat anti-mouse IgG(H+L)-HRP (Southern Biotechnologies). For the analysis of total IgG2c levels capture antibody goat anti-mouse Ig (H+L) and detection antibody goat anti-mouse IgG2c-biotin (Southern Biotechnologies), followed by streptavidin-HRP (Thermo Fisher Scientific) were used. Purified mouse IgM, IgG1, IgG2c, and IgG3 isotype control antibodies (BioLegend) were used as standards for the calculation of immunoglobulin concentrations. In the ELISA assays for the detection of PE-specific antibodies, the plates were coated with R-PE (10 μg/ml, ProZyme, Cedarlane), and developed with biotin goat anti-mouse IgM, IgG1, IgG2c, or IgG3 (Southern Biotechnologies), followed by streptavidin-HRP (Thermo Fisher Scientific). All the ELISAs were developed with SuperAquaBlue substrate (Thermo Fisher Scientific), and data acquired at 405 nm on EnSpire 2300 plate reader (PerkinElmer). Throughout the ELISA procedure, PBS with 0.05% Tween-20 was used as the Wash Buffer, PBS with 1% bovine serum albumin (Wisent) as the Blocking Buffer, and PBS with 0.1% bovine serum albumin (Wisent) as the Assay Diluent.

The detection of SRBC-specific antibodies was performed by flow cytometry, as described previously (62). Briefly, mouse serum samples were pre-diluted 1:60 in PBS and incubated with 3x 10^5^ SRBC for 30 min on ice. The SRBCs were washed and stained with goat anti-mouse IgG AlexaFluor488 (Poly4053, BioLegend, 1:200), or with goat anti-mouse IgM-biotin (1020-08, Southern Biotechnologies, 1:750), or IgG1-biotin (1070-08, Southern Biotechnologies, 1:750), followed by streptavidin – PerCP-Cy5.5 (BioLegend). Following thorough washing, the samples were analyzed on BD Fortessa and data processed with FlowJo software (Tree Star, BD Biosciences). SRBCs pre-incubated with serum of naive mice and stained as described above were analyzed as negative controls.

### Flow cytometry

Cell suspensions of mouse tissues were prepared in RPMI-1640 (Thermo Fisher Scientific) with 2% (v/v) FCS, 100μg/ml streptomycin and 100U/ml penicillin (Thermo Fisher Scientific). The cells were stained for surface-markers in PBS with 2% FCS for 20 min on ice, with the following antibodies, as summarized in **Supplementary Table S1**: Alexa Fluor 488 anti-GL7 (GL7, Biolegend); APC anti-CD21/CD35 (7E9, Biolegend), anti-CD86 (GL-1, Biolegend), anti-CD267/TACI (ebio8F10-3, eBioscience), and anti-IgD (11-26c.2a, BioLegend); APC-eFluor780 anti-CD45R/B220 (RA3-6B2, ThermoFisher); APC-Cy7 anti-CD5 (53-7.3, Biolegend); Brilliant UltraViolet 395 anti-CD43 (S7, BD Biosciences); Brilliant Violet 421 anti-CD95/Fas (Jo2, BD Biosciences) and anti-CD138 (281-2, Biolegend); Brilliant Violet 650 anti-CD45R/B220 (RA3-6B2, BioLegend); Biotin anti-IgG1 (polyclonal, 1070-08, Southern Biotech); eFluor450 anti-CD45R/B220 (RA3-6B2, Thermo Fisher); FITC anti-CD23 (B3B4, Invitrogen) and anti-Ki67 (SolA15, eBioscience); Pacific Blue anti-IgD (11-26c.2a, BioLegend); PE anti-Blimp-1 (5E7, Biolegend), anti-CD184/CXCR4 (L276F12, Biolegend) and anti-IgM (II/41, Thermo Fisher); PE-Cy7 anti-CD19 (6D5, BioLegend), anti-CD21/CD35 (eBio8D9, Invitrogen), and anti-CD38 (90, Invitrogen); PerCP-Cy5.5 anti-CD4 (RM4-4, Biolegend), anti-CD8a (53-6.7, Biolegend), anti-CD11b (M1/70, eBioscience), anti-CD19 (1D3, Tonbo Biosciences), anti-CD45R/B220 (RA3-6B2, BioLegend), anti-IgD (11-26c.2a Biolegend), anti-CD93 (AA4.1, Biolegend), anti-NK1.1 (PK136, Biolegend), and anti-TER119 (Ly-76, Biolegend). Brilliant Violet 785 Streptavidin (Biolegend) was used to detect biotin-conjugated antibodies. Viability Dye eFluor® 506 (eBioscience) was used to discriminate dead cells. Compensation was performed with BD CompBeads (BD Biosciences). The data were acquired on BD Fortessa and analyzed with FlowJo software (Tree Star, BD Biosciences).

### Cell isolation and sorting

Total B cells were isolated from mouse spleens via magnetic enrichment using with the EasySep Mouse CD19 Positive Selection Kit II (Stem Cell Technologies). For the FACS-sorting of B cell subsets, mouse spleens were mechanically dissociated in PBS with 0.1% BSA and 2mM EDTA, passed through 40 μm cell-strainers, and subjected to red blood cell lysis in ACK buffer (0.15M NH4Cl, 10mM KHCO3, 0.1mM EDTA). Cells were stained with the following antibodies, as summarized in **Supplementary Table S2**: Alexa Fluor 488 anti-GL7 (GL7, Biolegend), Brilliant Violet 421 anti-CD95/Fas (Jo2, BD Biosciences), and Brilliant Violet 650 anti-CD45R/B220 (RA3-6B2, BioLegend). Viability Dye 7-AAD (Biolegend) was used to discriminate dead cells. Sorting was performed on the FACSAria (BD Biosciences).

### Chromatin immunoprecipitation

ChIP was performed as described previously (49, 63). Cells were fixed with 1% formaldehyde in cell culture media for 10 min at room temperature, followed by addition of 0.125 M glycine to stop the fixation. Nuclei were extracted with 5 min lysis in 0.25% Triton buffer, followed by 30 minutes in 200mM NaCl buffer. Nuclei were resuspended in sonication buffer and sonicated for 12 cycles of 30 sec with a digital sonifier (Branson Ultrasonics) at 80%, with 30 sec rest in cooled circulating water. Beads immunocomplexes were prepared overnight by conjugating 40μL of Dynabeads Protein G (Thermo Fisher Scientific) with antibodies anti-BAP1 (Cell Signaling Technology, D1W9B, 52.8 μg) or anti-H2AK119ub (Cell Signaling Technology, D27C4, 5 μg). Immunoprecipitation was performed with an overnight incubation of the antibody-bead matrices with sheared chromatin from the equivalent of 5x10^6^ cells. Six washes were performed with low-stringency buffers, and samples de-crosslinked by overnight incubation in 1% SDS buffer at 65°C. Following treatments with RNaseA and Proteinase K, ChIP DNA was purified using Qiaquick PCR Cleanup kit (Qiagen).

ChIP-seq libraries were prepared using the Illumina TruSeq kit and sequenced on the Illumina NovaSeq 6000 in a paired-end 100bp configuration, with input DNA from the same cells sequenced as the negative controls. The reads were mapped to the UCSC mouse mm9 reference genome with Bowtie 1.0.0 (64), and BAP1 binding sites identified using the MACS1.4 peak detection algorithm (65), comparing for read enrichment against input DNA from the same cells. Normalized sequence read density profiles (bigwig) were generated with Homer (66) and visualized with IGV (67). Gene ontology (GO) enrichment analysis on the genes associated with the BAP1 ChIP-Seq binding clusters was performed on GREAT 4.0.4 (68) with Basal plus extension, searching for genes within 2kb upstream, 2kb downstream, or 200kb in distal.

### RNA sequencing

RNA-seq protocols were as previously described (49, 69). Briefly, RNA was isolated using the Mag-MAX total RNA kit (Ambion) and quality assessed using Bioanalyzer RNA Pico chips (Agilent). rRNA depletion and library preparation were performed using the SMARTer Stranded RNA-Seq kit (Takara Clontech). The libraries were sequenced on an Illumina Novaseq 6000 in a paired-end 100 bp configuration aiming for 50x10^6^ reads per sample. The quality of the sequencing reads was confirmed using the FastQC tool (Babraham Bioinformatics), and low-quality bases were trimmed from the read extremities using Trimmomatic v.0.33 (70). The reads were then mapped to the mouse UCSC mm9 reference genome assembly using Hisat2 v2.2.1 (71–73). Gene expression was quantified by counting the number of uniquely mapped reads with featureCounts using default parameters (74). We retained genes that had an expression level of at least 5 counts per 10^6^ reads (CPM) in at least 4 of the samples (75). TMM normalization and differential gene expression analyses were conducted using the edgeR Bioconductor package (76). Dimension reduction analysis was performed using the Principal Component and Partial Least Squares regression method (77). Pairwise comparisons were performed between genotypes or between treatments, and genes with changes in expression ≥ |2.0| fold and Benjamini-Hochberg adjusted *p* values ≤ 0.05 were considered significant. Gene ontology (GO) enrichment analyses on differentially expressed gene clusters were performed with DAVID Bioinformatics Resources 6.8 (78), and Gene Set Enrichment Analysis (GSEA) was performed with GSEA tool v4.3.2 using MSigDB v2022.1 with default configuration and permutation within gene sets (79). For the RNA-Seq data consolidation with the ChIP-Seq data, full gene annotations were obtained from UCSC mouse mm9 reference genome. An in-house Python script was developed to load the genomic locations of ChIP-Seq binding sites and RNA-Seq dysregulated genes, and search for gene TSS within a specific distance to each ChIP-Seq binding site, as previously described (80).

### Statistical analyses

Statistical analyses used Prism 9.5.1 (GraphPad Inc.), with Student’s *t*-test for two datasets and ANOVA for multiple comparisons, with further information is provided in Figure Legends.

## Results

### B-cell specific loss of BAP1 results in impaired antibody production

**Figure 1:**
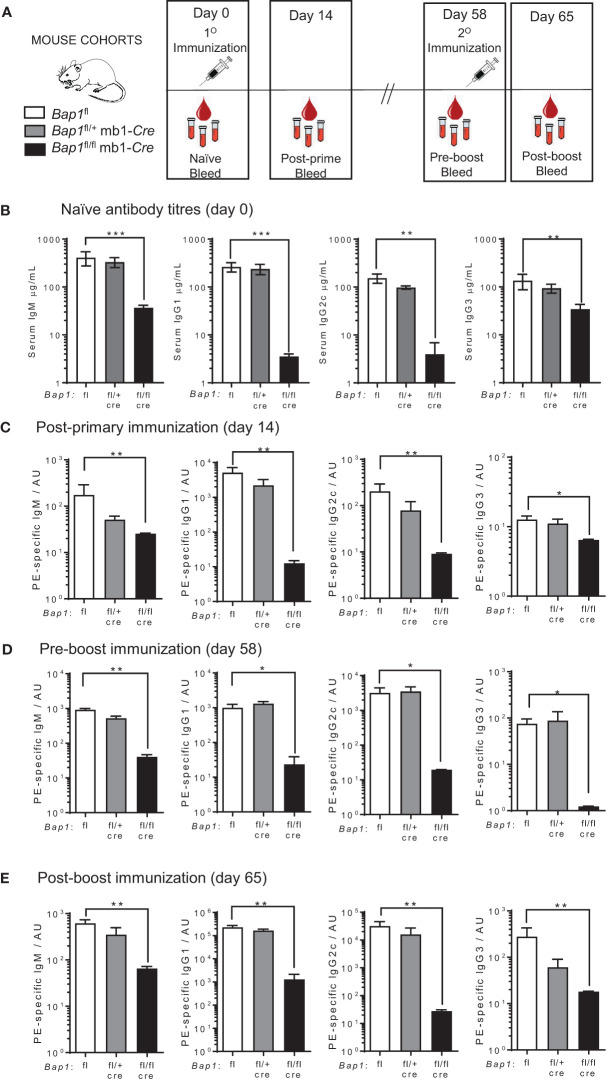
Reduced antibody levels and impaired antibody mediated immune response in *Bap1* ^fl/fl^ mb1-*Cre* mice. **(A)** Schematic of the experimental plan and timeline for the analyses of antibody production in *Bap1* ^fl/fl^ mb1-*Cre* and control *Bap1* ^fl^ and *Bap1* ^fl/+^ mb1-*Cre* mice. The mice received primary (day 0) and boost (day 58) immunizations of PE in CFA; post-primary immunization serum samples were acquired on day 14, pre-boost serum samples on day 58, and post-boost serum samples on day 65. **(B)** Levels of total IgM, IgG1, IgG2c, and IgG3 antibody isotypes in the serum of naïve mice of *Bap1* ^fl/fl^ mb1-*Cre* and control *Bap1* ^fl^ and *Bap1* ^fl/+^ mb1-*Cre* genotypes. The data for IgM, IgG1, and IgG3 is from 6-11 mice per genotype, and for IgG2c from 3-6 mice per genotype, consolidated from two independent experiments. **(C–E)** Antigen specific antibody titers in the mice of *Bap1* ^fl/fl^ mb1-*Cre* and control *Bap1* ^fl^ and *Bap1* ^fl/+^ mb1-Cre genotypes, immunized and bled as outlined in **(A)**. Data is from 3-6 mice per genotype. Bars represent means ± SEM; statistical analyses used non-parametric Kruskal-Wallis multiple comparison test with Dunn’s *post-hoc* test in GraphPad Prism 9.5.1; * *p*<0.05, ** *p*<0.01, *** *p*<0.001; AU - arbitrary units.

Due to the high expression of *Bap1* throughout the B cell lineage (**Supplementary Figure S1**) (50, 81) and its important role as a DUB for H2AK119ub in other cell types (28), we hypothesized that BAP1 may act as an important regulator of the transcriptional programs of B cell mediated immune response. In order to test this, we adopted the previously established *Bap1* ^fl/fl^ mb1-*Cre* mouse model, with the selective loss of BAP1 throughout the B cell lineage (49). We observed a strong reduction in total antibody titers in the serum of naïve *Bap1* ^fl/fl^ mb1-*Cre* mice, relative to control mice of *Bap1* ^fl/+^ and *Bap1* ^fl/+^ mb1-*Cre* genotypes, including both IgM antibodies and class-switched IgG1, IgG2c, and IgG3 antibodies (**Figures 1A, B**). We proceeded to challenge the mice with subcutaneous injections of phycoerythrin (PE) in CFA adjuvant and observed a strong reduction in the antigen specific antibody responses in *Bap1* ^fl/fl^ mb1-*Cre* relative to control mice, following both primary and boost immunizations, and affecting both IgM and class-switched IgG1, IgG2c and IgG3 isotypes (**Figures 1C–E**). Overall, this indicates the importance of BAP1 expression in the B cell lineage for the induction of B cell mediated immune response.

### Cell-intrinsic role of BAP1 in mature activated B cells in humoral immunity and antibody production

*Bap1* ^fl/fl^ mb1-*Cre* mice lack BAP1 expression throughout the B cell lineage, resulting in impaired B cell development and reduced B cell numbers in lymphoid organs (49), which could in part account for the impaired antibody production in these mice. Our objective therefore was to test the direct role of BAP1 in the regulation of B cell mediated immune response, independently of its functions in B cell development. For this purpose, we generated the *Bap1* ^fl/fl^ Cγ1-cre mice, with Cre expression driven from the IgG1 heavy chain locus and Cre activity restricted to the activated, germinal center (GC), and downstream B cell populations (55). In contrast to the *Bap1* ^fl/fl^ mb1-*Cre* mice (49), naïve *Bap1* ^fl/fl^ Cγ1-cre mice had normal numbers of splenic pre-GC B cells, including the transitional, follicular, and marginal zone subsets (**Supplementary Figure S2**). At the same time, the effective Cre-mediated *Bap1* ^fl^ to *Bap1* ^Δ^ allele conversion was demonstrated by PCR-genotyping of the genomic DNA from *ex vivo* stimulated *Bap1* ^fl/fl^ Cγ1-cre and control *Bap1* ^+/+^ Cγ1-cre B cells (**Supplementary Figures S3A, B**). Furthermore, RNA-seq analyses of splenic *Bap1* ^fl/fl^ Cγ1-cre GC B cells demonstrated a strong reduction in the transcript reads mapping to the *Bap1* floxed exons 6-12, as compared to the GC B cells from mice of control *Bap1* ^+/+^ Cγ1-cre genotype (**Supplementary Figure S3C, D**).

**Figure 2:**
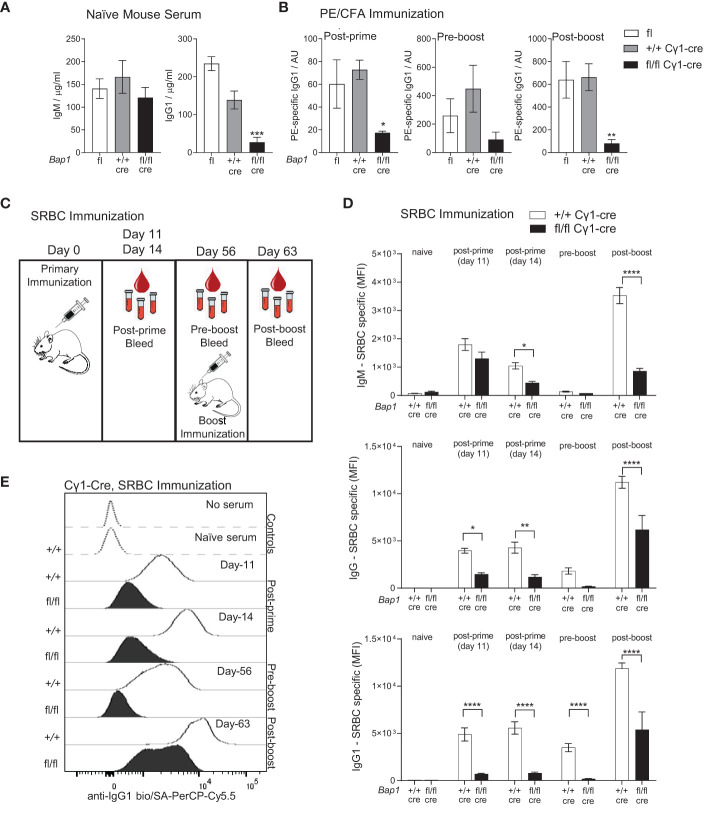
Reduced antibody levels and impaired antibody mediated immune response to immunization in *Bap1* ^fl/fl^ Cγ1-*Cre* mice. **(A)** Levels of total IgM and IgG1 antibody isotypes in the serum of naïve mice of *Bap1* ^fl/fl^ Cγ1-*Cre* and control *Bap1* ^fl^ and *Bap1* ^+/+^ Cγ1-*Cre* genotypes, assessed by ELISA. Data are from 9-11 mice per genotype, consolidated from two independent experiments. **(B)** Antigen specific antibody titers in the mice of *Bap1* ^fl/fl^ Cγ1-*Cre* and control *Bap1* ^fl^ and *Bap1* ^+/+^ Cγ1-Cre genotypes, immunized with PE in CFA adjuvant, as outlined above; data acquired with ELISA from 3-5 mice per genotype. **(C)** Experimental plan and timeline for the analyses of *Bap1* ^fl/fl^ Cγ1-*Cre* and control *Bap1* ^+/+^ Cγ1-*Cre* mouse responses to sheep red blood cell (SRBC) immunization. **(D)** Antigen specific antibody titers in the serum of mice of *Bap1* ^fl/fl^ Cγ1-*Cre* and control *Bap1* ^+/+^ Cγ1-Cre genotypes following SRBC immunization. Data are from 6-8 mice per genotype and reproduced in two independent experiments. Bars represent means ± SEM; statistical analyses used ANOVA with Sidak’s *post-hoc* test comparing *Bap1* ^fl/fl^ Cγ1-*Cre* and *Bap1* ^+/+^ Cγ1-Cre groups; * *p*<0.05, ** *p*<0.01, *** *p*<0.001, **** *p*<0.0001, or not significant if not indicated; AU – arbitrary units; MFI – mean fluorescence intensity. **(E)** Representative flow cytometry analyses of mouse serum for SRBC-binding antibody levels, involving the incubation of SRBCs with the serum followed by the staining of SRBCs for antibody binding, as previously described (62).

Cohorts of naïve *Bap1* ^fl/fl^ Cγ1-cre mice were analyzed for total serum antibody levels and demonstrated a significant reduction in IgG1 but not IgM titers relative to the *Bap1* ^+/+^ Cγ1-cre control group (**Figure 2A**). Further cohorts of *Bap1* ^fl/fl^ Cγ1-cre and control *Bap1* ^+/+^ Cγ1-cre mice were immunized, either subcutaneously with PE/CFA or intravenously with sheep red blood cells (SRBCs) and analyzed for antigen specific antibody titers over a time-course post-prime and post-boost immunization. A strong reduction in the antigen-specific antibody levels in *Bap1* ^fl/fl^ Cγ1-cre mice relative to the control *Bap1* ^+/+^ Cγ1-cre group was observed across both immunization models and most time-points (**Figures 2B–E**). Overall, these findings demonstrate the direct and cell intrinsic role of BAP1 in the induction of B cell mediated immune response, independent of its previously reported functions in B cell development (49).

### Germinal center dysfunction and plasma cell depletion with the loss of BAP1

**Figure 3:**
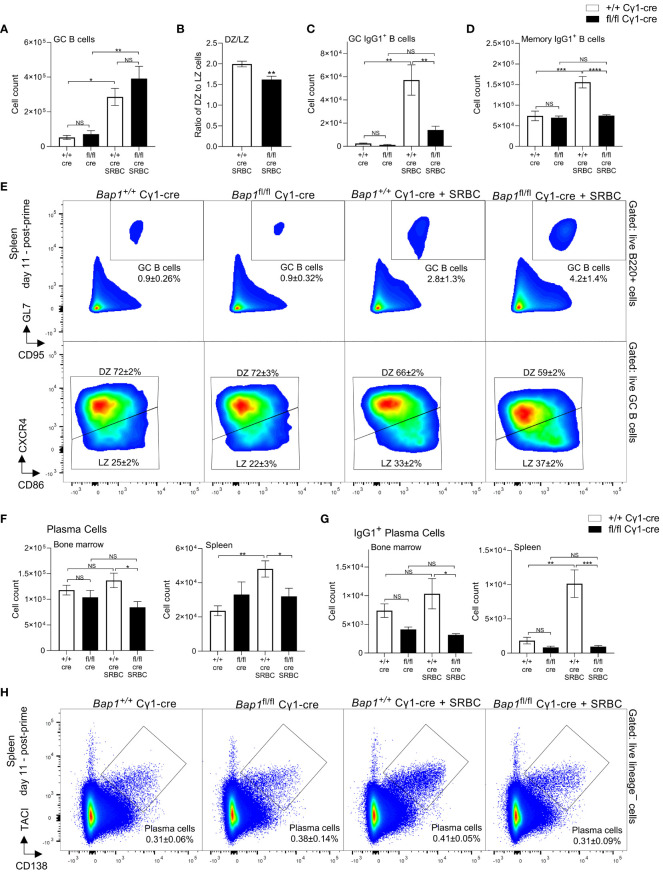
Analyses of germinal center (GC) B cells, memory B cells, and plasma cells in *Bap1* ^fl/fl^ Cγ1-*Cre* mice. Mice of *Bap1* ^fl/fl^ Cγ1-*Cre* and control *Bap1* ^+/+^ Cγ1-*Cre* genotypes, both naïve and at day 11 post-primary SRBC-immunization, were analyzed by flow cytometry. **(A)** Absolute numbers of GC B cells per mouse spleen, **(B)** ratio of dark zone (DZ) to light zone (LZ) GC B cells, **(C)** absolute numbers of IgG1^+^ GC B cells per mouse spleen, and **(D)** absolute numbers of IgG1^+^ memory B cells per mouse spleen, comparing between the *Bap1* genotypes and immunization conditions. **(E)** Representative flow cytometry plots analyzing splenic GC B cells; average percentage of cells in each gate relative to the parent gate for all the mice in each group is indicated as mean ± SD. **(F, G)** Absolute numbers of total plasma cells and IgG1^+^ plasma cells in the bone marrow and spleen, comparing between the *Bap1* genotypes and immunization conditions. **(H)** Representative flow cytometry analyses of plasma cells in the spleen of *Bap1* ^fl/fl^ Cγ1-*Cre* and control *Bap1* ^+/+^ Cγ1-*Cre* mice, both naïve and at day 11 post-primary SRBC-immunization; average percentage of cells in each gate relative to the parent gate for all the mice in each group is indicated as mean ± SD. Bars represent means ± SEM; statistical analyses used ANOVA with Sidak’s *post-hoc* test to compare between the *Bap1* ^fl/fl^ Cγ1-*Cre* and *Bap1* ^+/+^ Cγ1-Cre genotypes and immunization conditions; NS stands for not-significant; * *p*<0.05, ** *p*<0.01, *** *p*<0.001, **** *p*<0.0001. GC B cells were gated as live B220^+^GL7^+^CD95^+^ cells and divided into CXCR4^+^CD86^–^ dark zone (DZ) and CXCR4^–^CD86^+^ light zone (LZ) cell. Plasma cells were gated as live CD138 ^+^TACI ^+^ cells, negative for the lineage markers CD11b, TER119, CD4, CD8, and NK1.1.

To understand the impact of BAP1-loss on the cellular dynamics of humoral immune response, mice of *Bap1* ^fl/fl^ Cγ1-*Cre* and control *Bap1* ^+/+^ Cγ1-Cre genotypes, both naïve and challenged with SRBC-immunization (as shown in **Figure 2C**), were analyzed for the numbers of germinal center (GC) B cells, memory B cells, and plasma cells using flow cytometry (**Figure 3**). While there was no significant change in the absolute numbers of GC B cells in *Bap1* ^fl/fl^ Cγ1-*Cre* relative to control mice at day 11 following the SRBC-immunization (**Figure 3A**), we observed a significant reduction in the ratio of dark zone (DZ) to light zone (LZ) GC B cells (**Figure 3B**), primarily due to an expansion in the number of LZ B cells (data not shown). Importantly, *Bap1* ^fl/fl^ Cγ1-*Cre* mice showed a reduction in the numbers of class switched IgG1^+^ GC B cells (**Figure 3C**), with a significant depletion of IgG1^+^ cells within both the DZ and LZ subpopulations (data not shown), as well as a reduction in IgG1^+^ memory B cells (**Figure 3D**). Absolute numbers of plasma cells were also significantly reduced in both the spleen and bone marrow of *Bap1* ^fl/fl^ Cγ1-*Cre* mice (**Figure 3F**), with a particularly strong depletion of the IgG1^+^ class-switched plasma cells (**Figure 3G**). Further analysis of the plasma cell population demonstrated a more significant depletion of plasmablasts and early plasma cells as compared to late plasma cells in the *Bap1* ^fl/fl^ Cγ1-*Cre* mice (**Supplementary Figures S4A, B**), and a reduction in the proportion of plasma cells positive for the Ki-67 marker of cell proliferation at least in the bone marrow (**Supplementary Figure S4C**). Overall, the significant depletion of plasma cells likely contributes to the failure to produce and sustain normal antibody titers in the *Bap1* ^fl/fl^ Cγ1-*Cre* mice.

The mice were further analyzed for GC B cells, memory B cells, and plasma cells at day 7 after the boost SRBC immunization (**Supplementary Figure S5**). Surprisingly, an increase in GC B cells was observed in *Bap1* ^fl/fl^ Cγ1-*Cre* relative to control mice after the booster immunization (**Supplementary Figure S5A**), and this may reflect the modulatory effects of pre-existing high antibody titers on B cell recruitment into the GC in the control group (82). Furthermore, numbers of IgG1^+^ class-switched GC and memory B cells in the *Bap1* ^fl/fl^ Cγ1-*Cre* mice were restored to normal after the booster immunization (**Supplementary Figures S5C, D**), consistent with a considerable rise in their antibody titers at this time point (**Figures 2D, E**). However, the reduced ratio of DZ to LZ GC B cells and the significant depletion of class switched IgG1^+^ plasma cells persisted even after the booster immunization in *Bap1* ^fl/fl^ Cγ1-*Cre* mice (**Supplementary Figures S5B, G**). The persisting plasma cell phenotype likely contributes to the ongoing reduction in the antibody titers in *Bap1* ^fl/fl^ Cγ1-*Cre* mice.

### Loss of BAP1 in primary B cells does not impair antibody class switching

**Figure 4:**
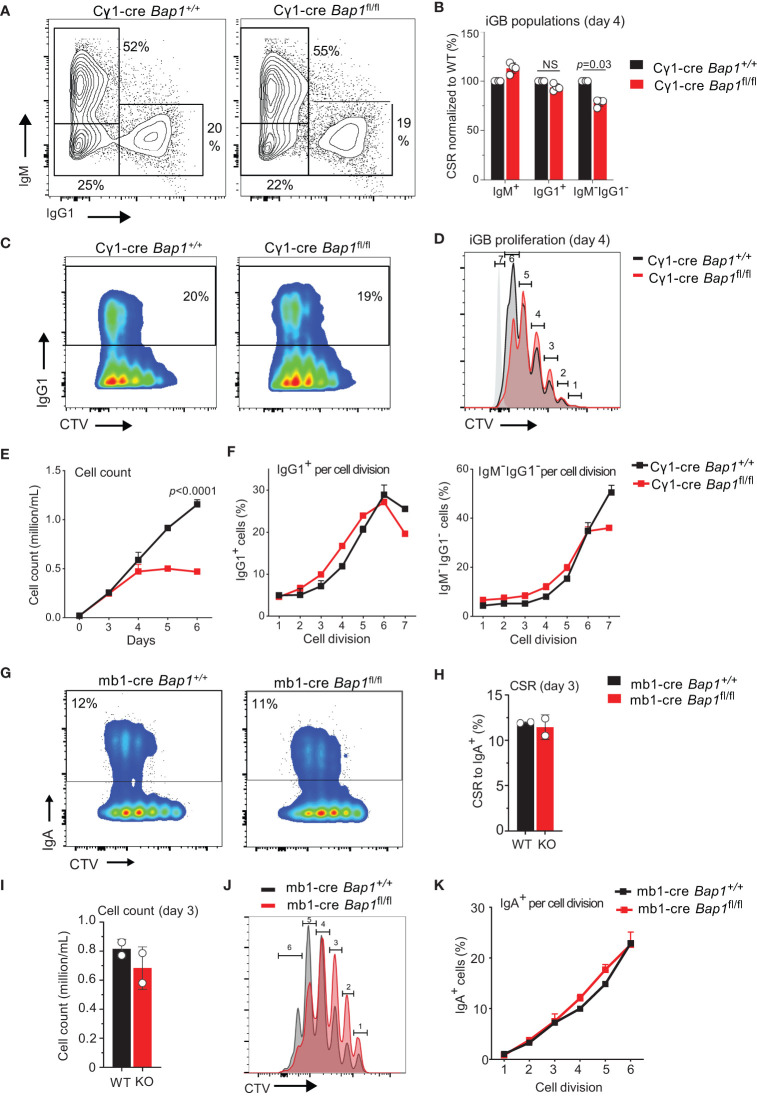
Loss of BAP1 does not impair immunoglobulin class switching in primary B cells. **(A–F)** Induced GC B cells (iGB) of *Bap1* ^fl/fl^ Cγ1-Cre and control *Bap1* ^+/+^ Cγ1-Cre genotypes were generated on 40LB feeders, as described previously (58, 60), and analyzed at day 4 of culture for **(A–C)** immunoglobulin class switching to IgG1, and **(C, D)** cell proliferation using the CellTrace Violet (CTV) dilution method. **(E)** Growth curves of iGBs of *Bap1* ^fl/fl^ Cγ1-Cre and control *Bap1* ^+/+^ Cγ1-Cre genotypes over a 6-day time course, evaluated with cell counting and demonstrating the impaired proliferation of *Bap1*-deficient B cells. **(F)** Antibody class switching per cell division, comparing *Bap1* ^fl/fl^ Cγ1-Cre and control *Bap1* ^+/+^ Cγ1-Cre iGBs at day 4 and showing no differences between the genotypes. Bars represent means ± SEM, with two mice per genotype. **(G–K)** Primary splenic B cells of *Bap1* ^fl/fl^ mb1-Cre and control *Bap1* ^+/+^ mb1-Cre genotypes were stimulated in culture to induce class switching to IgA. **(G)** Analyses of the cultures at day 3 of stimulation for class-switching to IgA and for cell proliferation using the CTV-dilution method demonstrated no differences in class switching between the *Bap1*-genotypes. **(H)** Quantification of the B cell cultures for class-switching to IgA demonstrated no differences between the *Bap1*-genotypes. **(I, J)** Analyses of B cell proliferation using **(I)** cell counting or **(J)** CTV-dilution method demonstrated a trend toward decreased proliferation of *Bap1* ^fl/fl^ mb1-Cre relative to the control *Bap1* ^+/+^ mb1-Cre B cells. **(K)** Antibody class switching per cell division comparing *Bap1* ^fl/fl^ mb1-Cre and control *Bap1* ^+/+^ mb1-Cre B cells showed no differences between the genotypes. Bars represent means ± SEM with two mice per genotype. Statistical analyses used **(B, H, I)** unpaired two-tailed Student’s *t*-test or **(E, K)** two-way ANOVA with Sidak’s multiple comparison test; *p*-values are indicated in the figure or not-significant (NS) if not indicated.

While the more significant impact of BAP1-loss on the numbers of IgG1^+^ rather than total GC B cells and plasma cells in the *Bap1* ^fl/fl^ Cγ1-*Cre* model may reflect the higher expression of Cre in the IgG1^+^ cells (55), it may also suggest a role for BAP1 in immunoglobulin class switching. We therefore proceeded to directly test the effects of *Bap1*-loss on antibody class switching *in vitro*. Induced GC B cells (iGBs) of *Bap1* ^fl/fl^ Cγ1-*Cre* and control *Bap1* ^+/+^ Cγ1-*Cre* genotypes were generated on the 40LB feeder cells that provide CD40L and BAFF, as described previously (58, 60). iGBs were analyzed at day 4 for class switching to IgG1 and demonstrated no significant differences between the *Bap1* ^fl/fl^ Cγ1-*Cre* and control *Bap1* ^+/+^ Cγ1-*Cre* cultures (**Figures 4A, B**). We further analyzed B cell proliferation in the iGBs cultures, using either the CellTrace Violet (CTV) dilution method with flow cytometry (**Figures 4C, D**, day 4) or manual cell counting (**Figure 4E**, days 0-6). This demonstrated a significant impairment in the proliferation for *Bap1* ^fl/fl^ Cγ1-*Cre* relative to control *Bap1* ^+/+^ Cγ1-*Cre* B cells under these culture conditions (**Figures 4C–E**). Further analyses of the antibody class switching rates per cell division in this model confirmed that the loss of *Bap1* does not directly interfere with class switching, and any reduction in class switched *Bap1* ^fl/fl^ Cγ1-*Cre* B cells can be attributed to impaired B cell proliferation (**Figures 4C, F**).

Primary B cells from *Bap1* ^fl/fl^ Cγ1-*Cre* and control *Bap1* ^+/+^ Cγ1-*Cre* mice were also stimulated in culture to class-switch to IgA with IL-21, anti-CD40, anti-IgM, TGF-β, and retinoic acid, as reported previously (59). Again, *Bap1* ^fl/fl^ Cγ1-*Cre* B cells demonstrated normal levels of class switching over 4 days in culture (**Supplementary Figures S6A, B**), though *Cre*-mediated *Bap1* ^fl^ to *Bap1* ^Δ^ allele conversion was observed as early as days 3, albeit without full loss of the *Bap1* ^fl^ allele (**Supplementary Figures S6C, D**). It is notable that no loss of cell proliferation was observed in these cultures, and this may reflect biological differences in the responses of *Bap1-*deficient B cells to different stimulation conditions or the lower efficiency of Cγ1-*Cre* in the cells undergoing class switching to IgA relative to IgG1, as shown by the persistent detection of the *Bap1* ^fl^ allele in these cells (**Supplementary Figure S6D**).

We further evaluated class switching using splenic B cells from *Bap1* ^fl/fl^ mb1-*Cre* and control *Bap1* ^+/+^ mb1-*Cre* mice, as in this model the Cre-mediated *Bap1*-allele deletion takes place from the early stages in B cell lineage development, and the full loss of BAP1 protein in *Bap1* ^fl/fl^ mb1-*Cre* splenic B cells has been validated through Western blotting in our previously published studies (49). Thus, to further assess the role of BAP1 in antibody class switching, *Bap1* ^fl/fl^ mb1-*Cre* and control *Bap1* ^+/+^ mb1-*Cre* B cells were stimulated in culture with IL-21, anti-CD40, anti-IgM, TGF-β, and retinoic acid to class-switch to IgA and analyzed at day 3 (**Figures 4G–K**). While *Bap1* ^fl/fl^ mb1-*Cre* B cells showed a trend toward impaired cell proliferation relative to the control B cells (**Figures 4G, I, J**), there were no differences in class switching per cell division between the *Bap1*-genotypes (**Figure 4K**), consistent with our findings with the *Bap1* ^fl/fl^ Cγ1-*Cre* B cells (**Figure 4F**, **Supplementary Figure S6B**). Overall, we conclude that BAP1 is not directly required for the normal progression of antibody class switching.

Given the impaired proliferation of *Bap1*-deficient B cells under some of the stimulation conditions presented above, we further analyzed the effects of BAP1 on B cell viability. No significant differences in cell viability were observed between *Bap1* ^fl/fl^ Cγ1-*Cre* and control *Bap1* ^+/+^ Cγ1-*Cre* germinal center B cells, memory B cells, or plasma cells, freshly isolated from naive mice or from mice at day 11 post-SRBC immunization (**Supplementary Figures S7A–D**). Further analysis of the plasma cell populations as plasmablasts, early plasma cells, and mature plasma cells also demonstrated no differences in viability based on the *Bap1*-genotype of the cells (data not shown). However, such analyses may not detect subtle changes in cell viability due to the rapid clearance of dead cells in healthy tissues, and we therefore further analyzed the viability of primary B cells of *Bap1* ^fl/fl^ Cγ1-*Cre* and control *Bap1* ^+/+^ Cγ1-*Cre* genotypes in culture, stimulated over 5 days with either anti-CD40 and IL-4, or with LPS and IL-4. This demonstrated a mild but statistically significant reduction in the viability of *Bap1* ^fl/fl^ Cγ1-*Cre* relative to control *Bap1* ^+/+^ Cγ1-*Cre* B cells under both stimulation conditions (**Supplementary Figure S7E**). Overall, we conclude that the loss of BAP1 can impair the viability of activated B cells.

### Impaired proliferation in Bap1 CRISPR/Cas9 gene targeted CH12F3 B cells

To further explore the roles and mechanisms of BAP1 activity in B cells, we targeted *Bap1* gene using CRISPR/Cas9 in the CH12F3 B cell lymphoma line (56), which is a commonly used model for the analyses of B cell activation, class switching, and associated cellular processes. gRNAs were designed to target *Bap1* exons 4 and 5 that encode the N-terminal catalytic domain of the protein (**Figure S8A**), and single-cell clones screened by PCR for deletions within the targeted regions of the *Bap1* locus. Further analyses of five *Bap1* ^Δ/Δ^ single-cell clones by Western blotting confirmed the full loss of BAP1 protein expression (**Supplementary Figure S8B**).

Based on the impaired proliferation of *Bap1* ^fl/fl^ Cγ1-*Cre* primary B cells in the iGB cultures (**Figure 4**) and on our previous report of impaired proliferation in *Bap1* ^fl/fl^ mb1-*Cre* pre-B cells (49), we analyzed the *Bap1* ^Δ/Δ^ CH12F3 cell clones for defects in proliferation, using the AlamarBlue resazurin/resorufin-based assay in bulk cell cultures, and the flow cytometry based CFSE dilution method. The assays demonstrated a significant reduction in cell proliferation for the *Bap1* ^Δ/Δ^ CH12F3 cell clones relative to control wild type CH12F3 cells (**Supplementary Figures S8C–E**), as well as the reduced viability of *Bap1* ^Δ/Δ^ CH12F3 cell at least under unstimulated culture conditions (**Supplementary Figure S8F**). This demonstrates the conservation of *Bap1*-deficiency phenotypes between CH12F3 cells and primary B cells and establishes the *Bap1* ^Δ/Δ^ CH12F3 cells as a model for further analyses of BAP1 functions in B cell biology.

### BAP1 genome-wide binding in B lymphocytes

**Figure 5:**
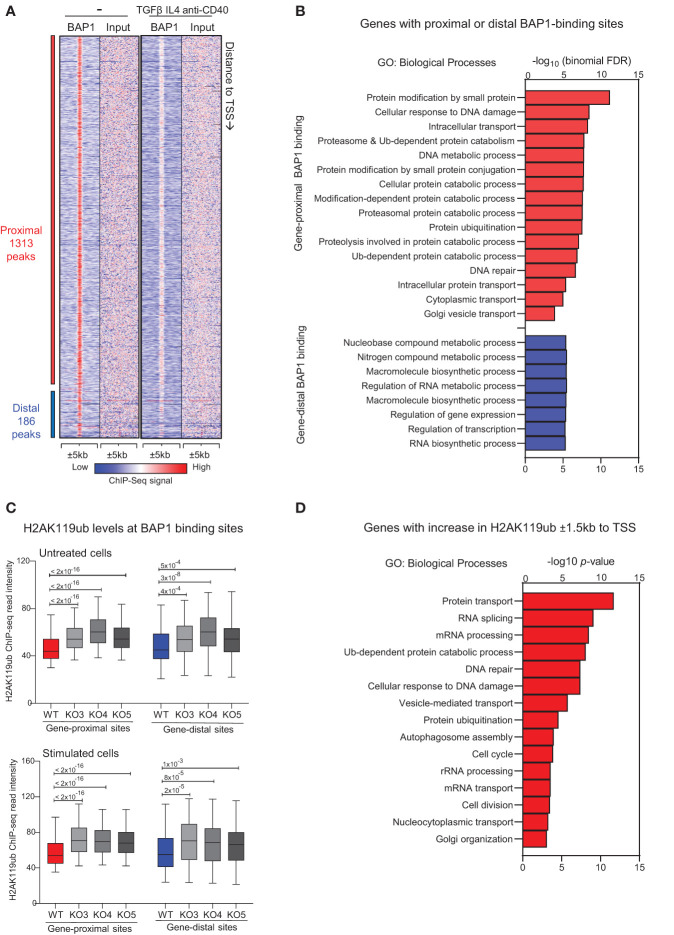
ChIP-seq analyses of BAP1 genome-wide binding and the effects of BAP1-loss on the histone H2AK119ub levels in CH12F3 B cells. **(A)** Heat-map showing the tag densities of 1,499 BAP1 binding sites identified in the ChIP-Seq experiments from wild type CH12F3 cells, untreated and at 72-hours of stimulation with TGFβ, IL-4, and anti-CD40. The sites are ranked based on their distance to the nearest gene transcription start site (TSS), and the gene-proximal sites are defined based on their location within 1kb to the nearest gene TSS. **(B)** Gene ontology (GO) analysis performed on the GREAT website, showing the biological process GO-terms enriched for the genes nearest to each BAP1 binding site, plotting the -log_10_(binomial FDR) for each GO-term. **(C)** ChIP-Seq analyses of histone H2AK119ub levels at the BAP1 binding sites in CH12F3 cells, including wild type (WT) control cells and three independent clones of CRISPR/Cas9 edited *BAP1*-knockout cells (KO3-4-5). Box plots show the H2AK119ub read intensities ±5kb to the gene-proximal and gene-distal BAP1 binding sites, comparing WT and KO cells; the *p*-values are calculated using Mann-Whitney U-test. **(D)** Gene ontology enrichment analyses on the genes that undergo an increase in the levels of histone H2AK119ub (fold change ≥ 2.0) around their transcription start sites ( ± 1.5kb to TSS) in *Bap1* ^Δ/Δ^ versus control CH12F3 B cells; the -log10(*p*-values) are listed for selected top GO-terms.

To identify the genomic regions directly regulated by BAP1 in mature B cells, we conducted ChIP-seq to map BAP1 genome-wide DNA-binding sites using an anti-BAP1 antibody and wild type CH12F3 cells, with and without stimulation with TGF-β, IL-4, and anti-CD40 over 72 hours. While there are limitations to using CH12F3 cells as a model of primary GC B cells, BAP1 is a challenging target, with most previously published ChIP-seq datasets using cells that express an epitope-tagged BAP1 protein (47, 83, 84). We have therefore also repeated the ChIP-seq analysis using CH12F3 cells stably expressing 3xFLAG-tagged BAP1 with an antibody against the FLAG epitope, as previously described (47, 49). We identified a total of 1,499 BAP1 binding sites (or peaks) across the genome (**Figure 5A**, **Supplementary Figure S9A**, **Supplementary Table S3A**), with the high concordance between the dataset from unstimulated and stimulated CH12F3 cells, indicating that BAP1 recruitment to chromatin is constitutive rather than induced with B cell stimulation. Among the BAP1 binding sites 88% were classified as gene-proximal based on their distance to the nearest gene transcription start site (≤1kb to TSS, **Supplementary Table S3A**). To gain insights into the biological functions of the BAP1 transcriptional target genes, ontology analyses were performed on the genes in the vicinity of the BAP1 binding sites using the Genomic Regions Enrichment of Annotations Tool (GREAT) (68). This demonstrated a highly significant association of the gene-proximal BAP1 binding sites with the genes involved in proteosome-dependent protein degradation, DNA damage response, and protein trafficking, and the association of the gene-distal BAP1 binding sites with the genes involved in the regulation of transcription and RNA processing (**Figure 5B**, **Supplementary Tables S3B, C**). This suggests the role of BAP1 in the regulation of many transcriptional programs essential for normal cell physiology, as well as for the induction of B cell activation and humoral immune response.

To compare the role of BAP1 as a transcriptional regulator in mature B cells to its role in other cell types, we consolidated our newly acquired BAP1 ChIP-Seq with the previously published BAP1 ChIP-Seq datasets from Ba/F3 pro-B cells (49), ES cells (85), and macrophages (47). This indicated a considerable overlap in BAP1 genomic binding and suggested shared BAP1 functions in the regulation of housekeeping transcriptional programs across many cell types (**Supplementary Figures S9A**, **Supplementary Tables S3D, E**). To gain further insights into the cross-talk between BAP1 and other transcriptional regulators, we also consolidated our BAP1 ChIP-Seq data with the public ChIP-Seq datasets for BAP1-associated transcriptional regulators, including HCF1 and OGT in macrophages (47), and ASXL1 in hematopoietic stem and progenitor cells (HSPC) (86). A significant overlap in the genomic localization was observed between BAP1 and BAP1-interacting proteins ASXL1, HCF1, and to a lesser extent OGT (**Supplementary Figure S9B**, **Supplementary Tables S3D, E**), indicating that BAP1 may act in cooperation with these transcriptional regulators in B cells, as in other cell types. Furthermore, we consolidated our BAP1 ChIP-seq data with the public ChIP-seq datasets from primary murine B cells for the other major regulators of the histone H2AK119ub epigenetic mark, including polycomb proteins RING1B, CBX7, YY1, EZH2 and deubiquitinase USP16 (18, 27). We observed an overlap in the genomic binding of BAP1 with components of the polycomb repressive complex 1 (PRC1), including its E3 ubiquitin ligase catalytic subunit RING1B (**Supplementary Figure S9B**, **Supplementary Table S3D**). This suggests that BAP1 and PRC1 may co-regulate histone H2AK119ub levels and turnover at shared genomic locations. Interestingly, we also observed an overlap in the genomic binding of BAP1 and another well-known H2AK119ub-deubiquitinase USP16 (**Supplementary Figure S9B**, **Supplementary Table S3D**), suggesting their cooperative or complementary functions.

### Non-redundant role of BAP1 as a deubiquitinase for histone H2AK119ub in B cells

To assess the role of BAP1 as a DUB for histone H2AK119ub in activated B cells, consistent with its reported function in other cell types (28), further ChIP-Seq analyses were performed to quantify the histone mark H2AK119ub. The study compared control wild type (WT) and BAP1-deficient *Bap1* ^Δ/Δ^ CH12F3 B cell lines, analyzing three independent clones of CH12F3 cells (KO3-4-5) and repeating the analyses at steady-state and following cell activation (TGFβ, IL-4, anti-CD40, 72 hours). ChIP-Seq data demonstrated that the loss of BAP1 resulted in a highly significant increase in histone H2AK119ub levels at the BAP1 binding sites and surrounding genomic regions ( ± 5kb) in all the three *Bap1* ^Δ/Δ^ clones and under both untreated and stimulated conditions (**Figures 5C**). This establishes the non-redundant role of BAP1 as a DUB for histone H2AK119ub in B lymphocytes.

Next, we set out to interrogate the biological functions of the genes regulated via the BAP1 DUB-activity for H2AK119ub. We therefore re-analyzed our ChIP-seq datasets to identify the genes with a >2-fold increase in H2AK119ub within ±1.5kb to their TSS in the *Bap1* ^Δ/Δ^ relative to control CH12F3 B cells (**Supplementary Table S4A**). GO-term analysis on this gene-set indicated that the genes undergoing an increase in H2AK119ub with the loss of BAP1 encode regulators of protein transport, Ub-dependent protein degradation, DNA damage response and repair, RNA processing, as well as autophagosome assembly and cell cycle progression (**Figure 5D**, **Supplementary Table S4B**). This demonstrates a strong concordance in the biological functions of this gene-set with the genes marked by BAP1-binding (**Figures 5B, D**). Overall, we conclude that BAP1 acts as a DUB for H2AK119ub in B cells to regulate many transcriptional programs essential for normal cell physiology, but also for B cell activation and the induction of humoral immune response.

For further insights into BAP1 functions as a DUB for H2AK119ub in the transcriptional networks of B cell mediated immune response, we performed supplementary analyses to consolidate our ChIP-seq datasets with the published RNA-seq data characterizing the transcriptional response of wild type CH12F3 cells to stimulation (87), and the transcriptional change with the transition from follicular to GC in primary B cells (81). In both cases, we identified a subset of differentially expressed genes that in our datasets have proximal BAP1 binding and sustain an increase in H2AK119ub with the loss of BAP1 (**Supplementary Tables S4D-F**). Such datasets may represent a starting point for future studies to validate and characterize genes under the direct transcriptional control of BAP1 in activated B cells.

### BAP1-regulated transcriptional programs of germinal center B cells

**Figure 6:**
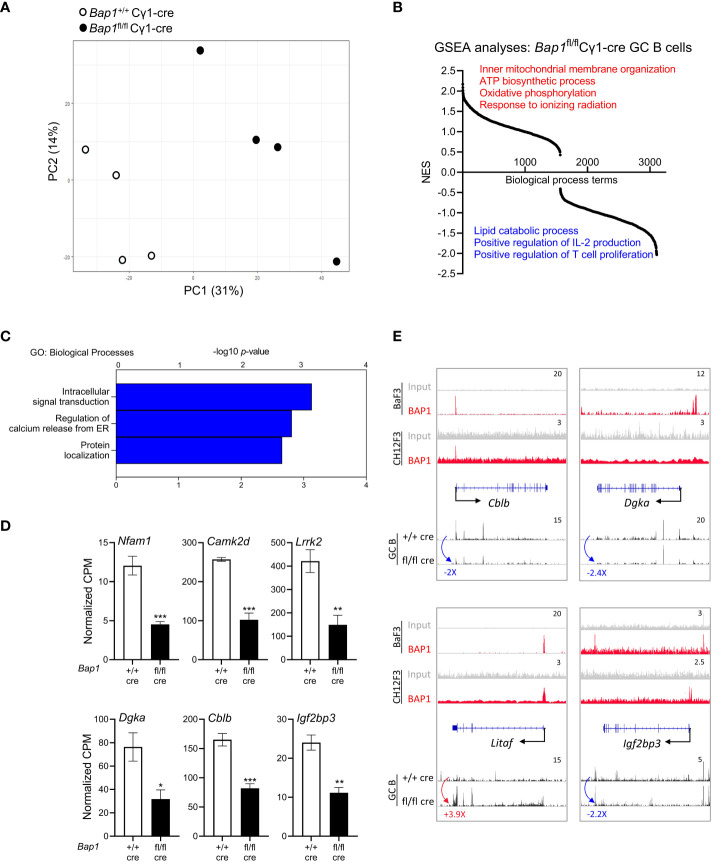
RNA-Seq analysis of the transcriptome of *Bap1* ^fl/fl^ Cγ1-*Cre* and control *Bap1* ^+/+^ Cγ1-Cre GC B cells. **(A)** Principal component analysis plot demonstrating the gene expression profiles of the RNA-Seq samples: differences between the *Bap1* genotypes are described by principal component 1 (PC1, 31% variability) and PC2 (14%). **(B)** Gene set enrichment analysis (GSEA) showing the normalized enrichment scores (NES) of 3,107 pre-established biological processes transcriptional signatures in *Bap1* ^fl/fl^ Cγ1-*Cre* relative to control *Bap1* ^+/+^ Cγ1-Cre GC B cells. **(C)** Gene ontology enrichment analysis of the genes downregulated in *Bap1* ^fl/fl^ Cγ1-*Cre* relative to control *Bap1* ^+/+^ Cγ1-Cre GC B cells, at fold change (FC) ≥ |2.0| and false discovery rate (FDR) ≤0.05; -log10(*p*-values) for the top GO-terms are presented. Equivalent analysis of the upregulated genes in provided in **Supplementary Table S5D**. **(D)** Examples of the genes downregulated in *Bap1* ^+/+^ Cγ1-Cre GC B cells and encoding important regulators on B cell receptor signaling, GC reaction, and humoral immunity; data presented as normalized read counts per million (CPM); bars represent mean ± SEM; * *p*< 0.05, ** *p*<0.01, *** *p*<0.001. **(E)** Genomic snapshots of selected genes representing the putative direct transcriptional targets of BAP1 in B cells, based on their altered expression in the *Bap1* ^fl/fl^ Cγ1-*Cre* GC B cells RNA-seq data and on BAP1 binding at their promoters in the ChIP-seq data. ChIP-Seq tracks for BAP1 and input DNA are shown on the top two lanes, including new data from CH12F3 B cells and previously published data from Ba/F3 pre-B cells (49). The gene feature track is shown in the middle, and averaged RNA-Seq tracks are in the bottom, with fold changes comparing expression in *Bap1* ^fl/fl^ Cγ1-*Cre* and control *Bap1* ^+/+^ Cγ1-Cre GC B cells indicated. The maximum data range of each track is indicated at the top-right corner.

To further analyze the role of BAP1 in the transcriptional regulation of B cell activation, RNA-Seq gene expression analysis was conducted on GC B cells isolated from the spleen of *Bap1* ^fl/fl^ Cγ1-cre and control *Bap1* ^+/+^ Cγ1-cre mice at day 11 post-SRBC immunization, with the cells gated for FACS-sorting as shown in **Supplementary Figure S10**. As already discussed, a strong reduction in the expression of the *Bap1* ^fl^ exons was demonstrated in the RNA-seq datasets from *Bap1* ^fl/fl^ Cγ1-cre relative to control GC B cells (**Supplementary Figures S3C, D**), confirming an effective Cre-mediated *Bap1* gene inactivation in the cells captured for the RNA-seq. Importantly, dimension reduction analysis of the gene expression profiles revealed a clear segregation of the *Bap1* ^fl/fl^ Cγ1-cre and control *Bap1* ^+/+^ Cγ1-cre GC datasets (**Figure 6A**, **Supplementary Table S5**, PC1 – 31% and PC2 –14% variance), which indicates a significant transcriptional change in GC B cells with the loss of BAP1.

To explore the biological functions of the genes dysregulated in *Bap1*-deficient GC B cells, gene set enrichment analysis (GSEA) was performed (79). This demonstrated a significant downregulation of the transcriptional signatures involved in B cell engagement with T-helper cells, such as ‘positive regulation of T cell proliferation’ and ‘positive regulation of IL2 production’ in the *Bap1* ^fl/fl^ Cγ1-cre GC B cells, as well as an upregulation of the transcriptional signatures linked to mitochondrial function such as ‘inner mitochondrial membrane organization’, ‘ATP biosynthetic process’, and ‘oxidative phosphorylation’ (**Figure 6B**, **Supplementary Table S5C**). We also conducted a differential gene expression analysis between the *Bap1* ^fl/fl^ Cγ1-cre and control *Bap1* ^+/+^ Cγ1-cre GC B cells, at the fold change (FC) ≥ |2.0| and false discovery rate (FDR) ≤ 0.05, which identified 94 upregulated and 76 downregulated genes in the *Bap1* ^fl/fl^ Cγ1-cre GC B cells (**Supplementary Table S5A**). Applying gene ontology (GO) analysis, we note the downregulation of the transcriptional signature of ‘intracellular signal transduction’ in the *Bap1* ^fl/fl^ Cγ1-cre GC B cells (**Figure 6C**, **Supplementary Table S5E**), with the downregulated genes including important positive and negative regulators of B cell receptor signaling, such as *Nfam1* (88), *Camk2d* (89), *Lrrk2* (90), *Dgka* (91), and *Cblb* (92, 93) (**Figure 6D**). As an example, *Cblb* encodes an E3 ubiquitin ligase that targets CD79a, CD79b, and IRF4 to regulate the initiation and progression of the GC-reaction (92, 93). Overall, the cell intrinsic loss of BAP1 in GC B cells resulted in a dysregulation of the transcriptional programs essential for normal induction of humoral immune response.

We consolidated the RNA-seq and ChIP-seq datasets to identify putative genes under the direct transcriptional control of BAP1 in B cells, as the genes dysregulated in *Bap1* ^fl/fl^ Cγ1-cre GC B cells and with BAP1-binding at their promoters in CH12F3 and/or BaF3 B cell lines. While our datasets showed no evidence for BAP1 regulation of the genes encoding the major mediators of B cell lineage choice, GC reaction, or plasma cell differentiation (*Bach2*, *Batf*, *Bcl6*, *Blimp1*/*Prdm1*, *Irf4*, *Irf8*, *Myc*, *Pax5*, *Xbp1*, **Supplementary Tables S3-S5**), we identified 73 putative BAP1-regulated genes (**Supplementary Tables S5F, G**). Such genes included the regulators of B cell receptor signaling *Dgka* and *Cblb*, already discussed above (**Figures 6D, E**) (91–93). The genes also included *Litaf* that encodes a repressor of *Bcl6*-expression and a regulator of autophagy and apoptosis in B cell lymphoma (94, 95), as well as *Igf2bp3* that encodes a reader of the N6-methyladenosine (m6A) RNA-modification, specifically induced in GC B cells and essential for normal induction of humoral immune response (**Figures 6D, E**) (96). In summary, our study identifies novel transcriptional targets of BAP1 that are essential for the progression of GC-reaction and can contribute to the failure of humoral immunity with the loss of BAP1 function.

## Discussion

Our study establishes the essential and cell-intrinsic role of the chromatin-binding DUB BAP1 in B cells for the normal induction of humoral immune response. It characterizes the impact of BAP1-loss on the genome-wide distribution of the H2AK119ub epigenetic mark and on the B cell transcriptome, and discovers novel BAP1-regulated genes with critical roles in the regulation of GC reaction and antibody-mediated immunity.

It is important to note that BAP1 is a multifunctional DUB with many putative substrates (28). Beyond histone H2AK119ub (19), BAP1 is reported to regulate the ubiquitination, stability, and activity of many transcription factors and enzymes at chromatin of various cell types, namely HCF1 (31, 32, 35), YY1 (35), FOXK1/2 (33, 34), KLF5 (36), PGC1α (37), and OGT (37, 47). BAP1 can also modulate the ubiquitination and stability of IP3R3, regulating Ca^2+^ release from endoplasmic reticulum and cell viability (38). Nevertheless, we demonstrate a major disruption in the H2AK119ub epigenetic landscape in *Bap1* ^Δ/Δ^ B cells, with an increase in H2AK119ub specifically at the BAP1 genome-binding sites. This establishes the major non-redundant role of BAP1 as a DUB for the H2AK119ub epigenetic mark in B cells and suggests this as the primary mechanism for the failure of B cell mediated immunity in the *Bap1* ^fl/fl^ Cγ1-cre model. Possible roles of other BAP1 substrates in B cell physiology remain to be further explored in future work.

Analyzing the effects of BAP1-loss on the transcriptome of GC B cells in consolidation with the BAP1 ChIP-seq data, we identified putative novel BAP1-regulated genes with essential roles in the GC reaction and humoral immunity. Furthermore, the significant overlap in BAP1 ChIP-seq genome binding sites in our B cell datasets with previously published datasets from other cell types indicated the engagement of BAP1 in shared housekeeping transcriptional programs. Although, due to the broad BAP1 functions in both housekeeping and cell-type specific transcriptional programs, we cannot attribute all the observed phenotypes to a defined set of BAP1-target genes, our analyses provide many novel BAP1-regulated genes the dysregulation of which can contribute to the failure of humoral immunity in the *Bap1* ^fl/fl^ Cγ1-cre model. This includes *Cblb* that encodes an E3 ubiquitin ligase known to target CD79a, CD79b, and IRF4 to regulate the GC-reaction (92, 93), as well as *Igf2bp3* that encodes a reader of the m6A RNA-modification, induced in GC B cells and essential for normal activation of humoral immunity (96). Compensatory activity of the YTHDF2 m6A-binding protein has been reported in *Igf2bp3*-deficient GC B cells (96), and may contribute to the overexpression of the transcriptional signatures of mitochondrial biogenesis in the *Bap1* ^fl/fl^ Cγ1-cre GC B cells. Downregulation of the transcriptional signatures of ‘IL2 production’ and ‘T cell proliferation’ in the *Bap1* ^fl/fl^ Cγ1-cre GC B cells is also notable, as failure to receive T cell help is known to result in mitochondrial dysfunction and apoptosis in activated B cells (97, 98). Another example of a novel BAP1-regulated gene identified in our work is *Litaf*, which encodes a repressor of *Bcl6*-expression and a regulator of autophagy in B cell lymphoma (94, 95), and indeed autophagy plays an important role in the regulation of GC reaction and plasma cell maintenance (99–102). While our study focused on the BAP1-regulated transcriptional programs in GC B cells, its functions in the downstream checkpoints in humoral immunity, such as in plasma and memory B cells, remains to be further explored. The reduction in plasma cell numbers and antibody titers in the *Bap1* ^fl/fl^ Cγ1-cre model is particularly notable, and our study did not quantify antigen specific plasma cells or assess the efficiency of *Bap1*-deletion within the plasma cell compartment. The roles and molecular mechanisms of BAP1 in plasma cell differentiation should be addressed in future work.

Loss of BAP1 in activated B cells in the *Bap1* ^fl/fl^ Cγ1-cre mouse model resulted in a severe defect in antibody production, and we noted the more profound reduction in IgG as compared to IgM isotypes. We hypothesized that this may reflect the enhanced Cγ1-cre activity in the B cells committed to undergo class switching to IgG1, or a direct role for BAP1 in the antibody class switching reaction. Indeed, epigenetic mechanisms are important across many checkpoints in antibody class switching, such as regulation of S-region accessibility and transcription, AID expression and targeting to chromatin, and in the DNA break-repair step (103). For instance, BAP1 was previously shown to facilitate DNA repair through the homologous recombination (HR) pathway in DT40 B cell lymphoma (39). However, we found normal class switching of *Bap1* ^fl/fl^ Cγ1-cre B cells when normalized to the significant reduction in B cell proliferation. This result is consistent with the major roles of the other repair pathways in antibody class switching, namely non-homologous end joining (NHEJ) and microhomology-mediated end joining (MMEJ) (104), and also rules out non-redundant roles of BAP1 in steps upstream from DNA damage in class switching. Nonetheless, BAP1 functions in the class switching reaction may be redundant with other chromatin associated DUBs (20), as for example the chromatin associated DUB USP22 was recently shown to promote DNA repair in antibody class switching (105).

Our current study focusses on the role of BAP1 specifically in mature activated B cells, with the *Bap1* ^fl/fl^ Cγ1-cre model allowing the selective analyses of its B cell-intrinsic functions in the induction of humoral immune response. However, beyond this experimental model, it is important to consider that BAP1 activities in other cell types may also contribute to the regulation of antibody mediated immunity *in vivo*. BAP1 is known to play an important cell-intrinsic role in T cell development in the thymus, as well as in the peripheral T cell expansion under homeostatic conditions and in response to antigenic stimulation (48). Furthermore, BAP1 activities in mesenchymal stromal cells (MSCs) are essential for normal B cell development in the bone marrow (106). Therefore, in addition to the cell intrinsic roles of BAP1 in B cells, elucidated in our current work, BAP1 functions in T cells and stromal compartments need to be considered when assessing its systemic roles in the regulation of humoral immunity *in vivo*, as for example in carriers of germline *BAP1* mutations.

Our study establishes the critical role of BAP1 as a transcriptional and epigenetic regulator in the GC reaction, which is a common site of cell transformation that gives rise to B cell lymphomas (4, 107). While BAP1 is a highly important tumor suppressor (28), lymphomas are not among the major cancer types associated with germline or acquired *BAP1* mutations, and our current study didn’t directly address the role of BAP1 in lymphomagenesis. Nevertheless, cases of non-Hodgkin lymphoma have been reported in carriers of germline *BAP1* mutations (108), and lymphomas with other genetic aberrations were shown to acquire *BAP1* gene silencing via epigenetic mechanisms (109). Therefore, we anticipate that future work in the *Bap1* ^fl/fl^ Cγ1-cre and other relevant murine models may further explore BAP1 functions as a tumor suppressor in B cell lymphoma.

In summary, our study provides novel insights into the epigenetic regulation of B lymphocyte biology and humoral immunity by BAP1, the major DUB for histone H2AK119ub, with potential implications for host responses to infections and vaccinations, and the mechanisms of B cell carcinogenesis.

## Data availability statement

RNA-Seq and ChIP-seq data are available in the National Center for Biotechnology Information GEO database under the following accession numbers: GSE244623 (NCBI tracking system #24319231) for RNA-seq and GSE245220 (NCBI tracking system #24330334) for ChIP-seq. All other data are available from the corresponding author upon request.

## Ethics statement

The animal study was approved by protocol MCGL-2011-6029 reviewed by the McGill Animal Care Committee. The study was conducted in accordance with the local legislation and institutional requirements.

## Author contributions

YL: Data curation, Formal Analysis, Investigation, Writing – original draft. HW: Supervision, Data curation, Formal analysis, Investigation, Writing – review & editing. NS: Formal analysis, Investigation, Writing – review & editing. YHL: Formal analysis, Investigation, Writing – review & editing. LT: Investigation, Writing – review & editing. JN: Conceptualization, Supervision, Writing – review & editing. DL: Conceptualization, Supervision, Writing – review & editing. AN: Conceptualization, Funding acquisition, Supervision, Writing – original draft, Formal analysis.
