# Supplementary material for: B-cell intrinsic regulation of antibody mediated immunity by histone H2A deubiquitinase BAP1
Source: Front Immunol. 2024 Mar 11;15:1353138. doi: 10.3389/fimmu.2024.1353138 (PMC10961346; doi:10.3389/fimmu.2024.1353138)

Supplemental Materials

B-cell Intrinsic Regulation of Antibody Mediated Immunity by Histone H2A Deubiquitinase BAP1

Yue Liang [1,2], HanChen Wang [1,2,3,4], Noé Seija [5,6], Yun Hsiao Lin [1,2], Lin Tze Tung [1,2,3,4], Javier M. Di Noia [5,6,7,8], David Langlais [2,3,4,8], Anastasia Nijnik[1,2,*]

[1] Department of Physiology, McGill University, Montreal, QC, Canada
[2] McGill University Research Centre on Complex Traits, McGill University, Montreal, QC, Canada
[3] McGill Genome Centre, Montreal, QC, Canada
[4] Department of Human Genetics, McGill University, Montreal, QC, Canada
[5] Institut de Recherches Cliniques de Montréal, Montreal, QC, Canada
[6] Molecular Biology Programs, Université de Montréal, Montreal, QC, Canada
[7] Department of Medicine, Université de Montréal, Montreal, QC, Canada
[8] Department of Microbiology and Immunology, McGill University, Montreal, QC, Canada

***Corresponding author:** Anastasia Nijnik, 368 Bellini Life Sciences Complex, 3649 Promenade Sir William Osler, McGill University, H3G 0B1 Montreal, QC, Canada. Tel: 1-514-398-5567, Fax: 1-514-398-2603, Email: anastasia.nijnik@mcgill.ca.

**Contents**	**Pages**
Supplemental Tables S1 and S2: flow cytometry antibodies and gating methods	2-4
Legends of Supplemental Tables S3, S4, and S5: ChIP-seq and RNA-seq data summaries *with the Supplemental Tables S3, S4, and S5 included as separate excel documents*	5-6
Supplemental Figures S1-S10 with Legends	7-26
References	27
Graphical Abstract	28

SUPPLEMENTAL METHODS TABLES

Table S1A. Antibodies and other reagents used in the flow cytometry analyses of mouse tissues.

Fluorophore	Target	Manufacturer	Catalog Number	Clone
AF488	GL7	BioLegend	144612	GL7
APC	CD21/CD35	Biolegend	123412	7E9
APC	CD86	BioLegend	105012	GL-1
APC	CD267/TACI	Invitogen	17-5942-82	ebio8F10-3
Biotin	IgG1	Southern Biotech	1070-08	polyclonal
BV421	CD138	BioLegend	142508	281-2
BV421	CD95/Fas	BD Bioscience	562633	Jo2
BV650	CD45R/B220	BioLegend	103241	RA3-6B2
BV785	Streptavidin	BioLegend	405249	n/a
FITC	KI67	eBioscience	11-5698-80	SolA15
FITC	CD23	Invitrogen	A15820	B3B4
Pacific Blue	IgD	BioLegend	405712	11-26c.2a
PE	Blimp-1	BioLegend	150005	5E7
PE	CD184/CXCR4	BioLegend	146505	L276F12
PE	IgM	Invitogen	12-5790-83	II/41
PE-Cy7	CD19	BioLegend	115520	6D5
PE-Cy7	CD21/CD35	Invitogen	13-0211-82	eBio8D9
PE-Cy7	CD38	Invitogen	25-0381-82	90
PerCPCy5.5	CD11b	eBioscience	45-0112-82	M1/70
PerCPCy5.5	CD4	BioLegend	116012	RM4-4
PerCPCy5.5	CD8a	BioLegend	100734	53-6.7
PerCPCy5.5	IgD	BioLegend	405709	11-26c
PerCPCy5.5	NK1.1	BioLegend	108727	PK136
PerCPCy5.5	Ter119	BioLegend	116228	Ly-76
PerCPCy5.5	CD19	Tonbo Biosciences	50-105-4943	1D3
PerCPCy5.5	CD45R/B220	BioLegend	103236	RA3-6B2
PerCPCy5.5	CD93	BioLegend	136512	AA4.1
eFluor506	Viability Dye	eBioscience	65-0866-18	n/a

Table S1B. Flow cytometry gating strategies to identify B cell subpopulations in mouse tissues.

**Cell Subsets**	**Markers used for gating**
B cells	$B220^{+}$
Memory B cells	$B220^{+}$, $CD38^{+}$, $GL7^{–}$
Germinal centre (GC) B cells	$B220^{+}$, $CD95/Fas^{+}$, $GL7^{+}$
DZ GC B cells	$B220^{+}$, $CD95/Fas^{+}$, $GL7^{+}$, $CD86^{–}$, $CXCR4^{+}$
LZ GC B cells	$B220^{+}$, $CD95/Fas^{+}$, $GL7^{+}$, $CD86^{+}$, $CXCR4^{–}$
Plasmablasts/Plasma cells	$Lin^{–}$ ($CD11b^{–}$, $TER119^{–}$, $CD4^{–}$, $CD8^{–}$, $NK1.1^{–}$), $CD138^{+}$, $TACI^{+}$
Plasmablast	$Lin^{–}$ ($CD11b^{–}$, $TER119^{–}$, $CD4^{–}$, $CD8^{–}$, $NK1.1^{–}$), $CD138^{+}$, $TACI^{+}$, $CD19^{+}$, $B220^{+}$
Early Plasma cells	$Lin^{–}$ ($CD11b^{–}$, $TER119^{–}$, $CD4^{–}$, $CD8^{–}$, $NK1.1^{–}$), $CD138^{+}$, $TACI^{+}$, $CD19^{+}$, $B220^{–}$
Mature Plasma cells	$Lin^{–}$ ($CD11b^{–}$, $TER119^{–}$, $CD4^{–}$, $CD8^{–}$, $NK1.1^{–}$), $CD138^{+}$, $TACI^{+}$, $CD19^{–}$, $B220^{–}$
Plasmablast	$Lin^{–}$ ($CD11b^{–}$, $TER119^{–}$, $CD4^{–}$, $CD8^{–}$, $NK1.1^{–}$), $CD138^{+}$, $TACI^{+}$, $CD19^{+}$, $B220^{hi}$, $Ki67^{+}$
Early Plasma cells	$Lin^{–}$ ($CD11b^{–}$, $TER119^{–}$, $CD4^{–}$, $CD8^{–}$, $NK1.1^{–}$), $CD138^{+}$, $TACI^{+}$, $Blimp1^{+}$, $B220^{lo}$, $Ki67^{–}$
Late Plasma cells	$Lin^{–}$ ($CD11b^{–}$, $TER119^{–}$, $CD4^{–}$, $CD8^{–}$, $NK1.1^{–}$), $CD138^{+}$, $TACI^{+}$, $Blimp1^{+}$, $B220^{–}$, $Ki67^{–}$
T1 Transitional B cells	$CD19^{+}$, $B220^{+}$, $CD93^{+}$, IgM^{+}, CD23-
T2 Transitional B cells	$CD19^{+}$, $B220^{+}$, $CD93^{+}$, IgM^{+}, $CD23^{+}$
T3 Transitional B cells	$CD19^{+}$, $B220^{+}$, $CD93^{+}$, IgM^{lo}, $CD23^{+}$
Follicular (FO) B cells	$CD19^{+}$, $B220^{+}$, $CD93^{–}$, IgM^{+}, $CD21^{+}$, IgD^{+}
FO I B cells	$CD19^{+}$, $B220^{+}$, $CD93^{–}$, IgM^{lo}, $CD21^{+}$, IgD^{+}
FO II B cells	$CD19^{+}$, $B220^{+}$, $CD93^{–}$, IgM^{hi}, $CD21^{+}$, IgD^{+}
Marginal Zone (MZ) B cells	$CD19^{+}$, $B220^{+}$, $CD93^{–}$, IgM^{hi}, $CD21^{hi}$, $CD23^{lo}$
MZ Precursor (MZP) B cells	$CD19^{+}$, $B220^{+}$, $CD93^{–}$, IgM^{hi}, $CD21^{hi}$, $CD23^{hi}$
B1a B cells	$CD19^{+}$, $B220^{lo}$, $CD43^{+}$, $CD5^{+}$
B1b B cells	$CD19^{+}$, $B220^{lo}$, $CD43^{+}$, $CD5^{-}$

Table S2A. Flow cytometry antibodies and other reagents used for FACS cell sorting.

**Fluorophore**	**Target**	**Manufacturer**	**Catalog Number**	**Clone**
AF488	GL7	BioLegend	144612	GL7
BV421	CD95/Fas	BD Bioscience	562633	Jo2
BV650	CD45R/B220	BioLegend	103241	RA3-6B2
7-AAD	Viability Dye	Biolegend	420404	n/a

Table S2B. Flow cytometry antibodies and other reagents used for FACS cell sorting.

Cell Subsets	Markers used for gating
GC B cells	$B220^{+}$, $CD95/Fas^{+}$, $GL7^{+}$

Legends of Supplemental Data Tables

Supplemental Data Tables S3-S5 are attached as separate excel files.

Table S3. ChIP-seq of BAP1 genome-wide binding sites in CH12F3 B cells; (related to Figure 5). **(A)** List of all BAP1 ChIP DNA-binding sites (peaks) identified in CH12F3 B cells, with the following information provided for each peak: peak number that corresponds to the row on the heatmap in Figure 5A; peak genomic location; distance to the nearest gene transcription start site (TSS); origin of the peak in the BAP1-WT or BAP1-3xFLAG ChIP dataset with or without cell stimulation; peak group as either gene proximal (<1kb to TSS) or gene distal (>1kb to TSS); the identity of the nearest gene; tag count in each individual ChIP sample corresponding to the normalized read intensity ±100bp around the peak summit. **(B-C)** Gene ontology enrichment analysis on the genes nearest to each BAP1 DNA-binding peak, performed using GREAT 4.0.4 with Basal plus extension, searching for genes within 2kb upstream, 2kb downstream, or 200kb in distal to each peak. The analyses were performed separately for **(B)** gene-proximal BAP1 binding sites (<1kb to TSS), and **(C)** gene-distal BAP1 binding sites (>1kb to TSS). The -log10(binomial FDR) values are listed for each GO term; (related to Figure 5B). **(D-E)** Consolidation of our new BAP1 ChIP-seq data from CH12F3 B cells with public BAP1 ChIP-seq datasets from Ba/F3 pre-B cells (1), macrophages (2), and embryonic stem cells (3), as well as with ChIP-seq datasets for BAP1-associated transcriptional regulators, including HCF1 and OGT in macrophages (2), and ASXL1 in hematopoietic stem and progenitor cells (4), and with ChIP-seq datasets for other regulators of histone H2AK119ub, including polycomb proteins RING1B, CBX7, YY1, EZH2 and deubiquitinase USP16 (5, 6). The public datasets were downloaded and re-analyzed using our pipeline; all data are from mouse. **(D)** The information provided for each BAP1 peak includes: the dataset(s) of origin for the peak; the identity of the nearest gene; tag count in each individual ChIP sample, corresponding to normalized read intensity ±100bp around the peak summit. **(E)** BAP1 binding peaks were classified based on their presence or absence in the ChIP datasets from B cells, macrophages, and/or ES-cells, and then GREAT analysis was performed for the genes closest to each set BAP1 peaks, as described in (B-C), to evaluate their biological functions. The -log10(binomial FDR) values are listed for each GO term.

Table S4. H2AK119ub ChIP-seq analyses in CH12F3 B cells: the impact of BAP1 loss on the genome-wide levels of histone H2AK119ub at gene transcription start sites; (related to Figure 5). **(A)** Identifying the genes that undergo a change in the levels of histone H2AK119ub around their transcription start sites (±1.5kb to TSS) in *Bap1*$^{\Delta/\Delta}$ versus control CH12F3 B cells. Overall, 1,867 genes in untreated and 1,433 genes in activated CH12F3 B cells that undergo a change in H2AK119ub ≥ |2.0| were identified. Information provided for each gene includes: the gene symbol, name, and genomic location; tag counts in each H2AK119ub ChIP sample corresponding to the normalized read intensity ±1.5kb to TSS; and calculations for the fold change in H2AK119ub levels with the loss of BAP1. **(B-C)** Gene ontology enrichment analyses on the genes that undergo a change in the levels of histone H2AK119ub around their transcription start sites (±1.5kb to TSS) in *Bap1*$^{\Delta/\Delta}$ versus control CH12F3 B cells (fold change ≥ |2.0|), with **(B)** showing the GO-terms enriched for the genes with an increase in H2AK119ub, and **(C)** showing the GO-terms enriched for the genes with a decrease in H2AK119ub. The -log10(*p*-values) are listed for each GO term. **(D)** Consolidation of the BAP1 and H2AK119ub ChIP-seq datasets from CH12F3 B cells from the current study with the previously published RNA-seq data characterizing the response of wild type CH12F3 B cells to stimulation (GSE118794; TGF-β, IL-4, anti–

CD40) (7). The analysis highlights the subset of genes that undergo a significant change in expression in wild type CH12F3 cells in response to stimulation (GSE118794, TGF-β, IL-4, anti–CD40) (7), and in our ChIP-seq data have a BAP1 binding site/peak within 5kb to the gene TSS, and undergo an increase in H2AK119ub levels ≥1.5-fold in at least one of the BAP1-KO relative to control wild type CH12F3 B cell clones. **(E)** Consolidation of the H2AK119ub ChIP-seq datasets from CH12F3 B cells from the current study with the previously published RNA-seq characterizing the transcriptional changes with transition from follicular (FO) to germinal centre (GC) in primary mouse B cells (*www.immgen.org*) (8). The analysis highlights the subset of genes that undergo a significant change in expression with GC B cell differentiation (FC≥±1.5, FDR≤0.01, *www.immgen.org*) (8), and in our ChIP-seq data undergo a change in H2AK119ub levels ≥2-fold in at least one of the BAP1-KO relative to control wild type CH12F3 clones. **(F)** Gene ontology enrichment analysis on the genes from (E) that undergo a significant change in expression with GC B cell differentiation (FC≥±1.5, FDR≤0.01, *www.immgen.org*) (8), and in our ChIP-seq data undergo a change in H2AK119ub levels ≥2-fold in at least one of the BAP1-KO relative to control wild type CH12F3 clones. The -log10(binomial FDR) values are listed for each GO term in columns B-I, with the corresponding dysregulated genes listed in columns J-Q. The analysis shows that the gene set upregulated in GC versus FO mouse primary B cells and undergoing an increase in H2AK119ub levels in BAP1-KO CH12F3 B cells is enriched for GO-terms related to cell cycle progression, DNA damage response and repair, ubiquitin-dependent protein degradation, autophagy, and lymphocyte proliferation.

Table S5. RNA-seq transcriptional analysis of germinal center (GC) B cells sorted from the spleen of *Bap1*$^{fl/fl}$ Cγ1-cre and control *Bap1*$^{+/+}$ Cγ1 mice; (related to Figure 6).

(A) The list of genes differentially expressed between *Bap1*$^{fl/fl}$ Cγ1-cre and control *Bap1*$^{+/+}$ Cγ1-cre GC B cells, at fold change (FC) ≥ |2.0| and false discovery rate (FDR) ≤0.05. Information provided for each gene includes full gene name, fold change, false discovery rate (FDR), whether the gene is up-regulated or down-regulated in *Bap1*$^{fl/fl}$ Cγ1-cre GC B cells, and normalized read counts per million (CPM). **(B)** Full list of genes expressed in GC B cells from *Bap1*$^{fl/fl}$ Cγ1-cre and control *Bap1*$^{+/+}$ Cγ1-cre mice. **(C)** Normalized enrichment scores (NES) of biological process gene expression signatures used in the gene set enrichment analysis (GSEA). The comparison is between *Bap1*$^{fl/fl}$ Cγ1-cre and control *Bap1*$^{+/+}$ Cγ1-cre cells, where *Bap1*$^{+/+}$ Cγ1-cre is the baseline. Positive values indicate upregulation in *Bap1*$^{fl/fl}$ Cγ1-cre GC B cells and negative values indicate downregulation in *Bap1*$^{fl/fl}$ Cγ1-cre GC B cells. **(D-E)** Gene ontology enrichment analysis showing select enriched biological process GO-terms for the genes **(D)** up-regulated and **(E)** down-regulated in *Bap1*$^{fl/fl}$ Cγ1-cre GC B cells compared to control *Bap1*$^{+/+}$ Cγ1-cre GC B cells, at fold change (FC) ≥ |2.0| and false discovery rate (FDR) ≤0.05. **(F-G)** Consolidation of the RNA-seq transcriptional analysis of *Bap1*$^{fl/fl}$ Cγ1-cre GC B cells with ChIP-seq analysis of BAP1 genome-wide binding sites in **(F)** BaF3 pre-B cells and **(G)** CH12F3 B cells. Full gene annotations with transcription start sites (TSS) were obtained from the UCSC mouse mm9 reference genome, and an in-house R script was developed to load the genomic locations of ChIP-seq binding sites and the TSS locations of RNA-Seq dysregulated genes, and to search for gene TSS located within 5kb to each BAP1 ChIP-seq binding site. **(F)** List of genes with dysregulation in the RNA-seq transcriptional analysis of *Bap1*$^{fl/fl}$ Cγ1-cre GC B cells and a BAP1 binding site within 5kb to the gene TSS in BaF3 pre-B cell line (1). **(G)** List of genes with dysregulation in the RNA-seq transcriptional analysis of *Bap1*$^{fl/fl}$ Cγ1-cre GC B cells and a BAP1 binding site within 5kb to the gene TSS in CH12F3 B cell line.

Supplemental Figure Legends

Figure S1. High expression of *Bap1* gene throughout the B cell lineage. Normalized expression of *Bap1* gene and housekeeping genes *Gapdh* and *Hprt* in hematopoietic stem cells, progenitor cells, and across the B cell lineage. RNA-Seq data from wild type C57BL/6J mice was retrieved from the ImmGen datasets (8), and RNA-Seq data analyses, normalization, and quantification of gene expression were performed as previously described (9). Abbreviations: HSC – hematopoietic stem cells, MPP1-4 – multipotent progenitor cells 1-4, CLP – common lymphoid progenitor, pre-pro and pro – pre-pro-B and pro-B cells, FO – splenic follicular B cells, MZ – splenic marginal zone B cells, GC – germinal centre B cells, PC – plasma cells.

Figure S1

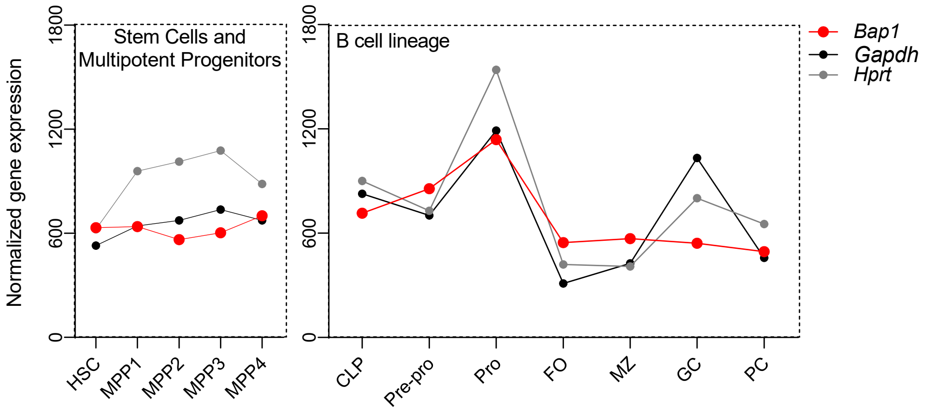

Normalized gene expression
0
600
1200
1800
Stem Cells and
Multipotent Progenitors
HSC
MPP1
MPP2
MPP3
MPP4
B cell lineage
CLP
Pre-pro
Pro
FO
MZ
GC
PC
Bap1
Gapdh
Hprt

Figure S2. Analysis of splenic B cell populations in naïve *Bap1*$^{fl/fl}$ Cγ1-cre mice. (A-B) Quantification of transitional T1, T2, and T3, follicular FOL I and FOL II, marginal zone progenitor (MZP) and marginal zone (MZ) B cell population in the spleen of *Bap1*$^{fl/fl}$ Cγ1-cre and control *Bap1*$^{+/+}$ Cγ1-cre mice, presented **(A)** as a percentage of live CD19^{+} B cell lineage splenocytes, and **(B)** as an absolute cell number per mouse spleen. Cells are gates as live CD19^{+} B cells, followed by B220^{+}CD93^{+}IgM^{+}CD23$^{–}$ for T1, B220^{+}CD93^{+}IgM^{+}CD23^{+} for T2, and B220^{+}CD93^{+}IgM^{lo}CD23^{+} for T3 transitional B cells, B220^{+}CD93$^{–}$CD21^{+}IgM^{+}IgD^{+} for FOL I and B220^{+}CD93$^{–}$CD21^{+}IgM^{hi}IgD^{+} for FOL II follicular B cells, B220^{+}CD93$^{–}$CD21^{hi}IgM^{hi}CD23^{+} for MZP and B220^{+}CD93$^{–}$CD21^{hi}IgM^{hi}CD23$^{–}$ for MZ B cells, as previously described (10). Data are from 4 mice per genotype. Bars represent mean ± SEM; statistical analysis by *t*-test; ** $p<0.01$; not significant if not indicated. **(C)** Representative flow cytometry plots of the spleen of naïve *Bap1*$^{fl/fl}$ Cγ1-cre and control *Bap1*$^{+/+}$ Cγ1-cre mice, showing the gating for T1, T2, T3, FOL I, FOL II, MZP, and MZ B cell populations, according to the markers listed above. Percentages of cells within each gate relative to the parent gate for each mouse genotype are presented as mean ± S.D.

Figure S2

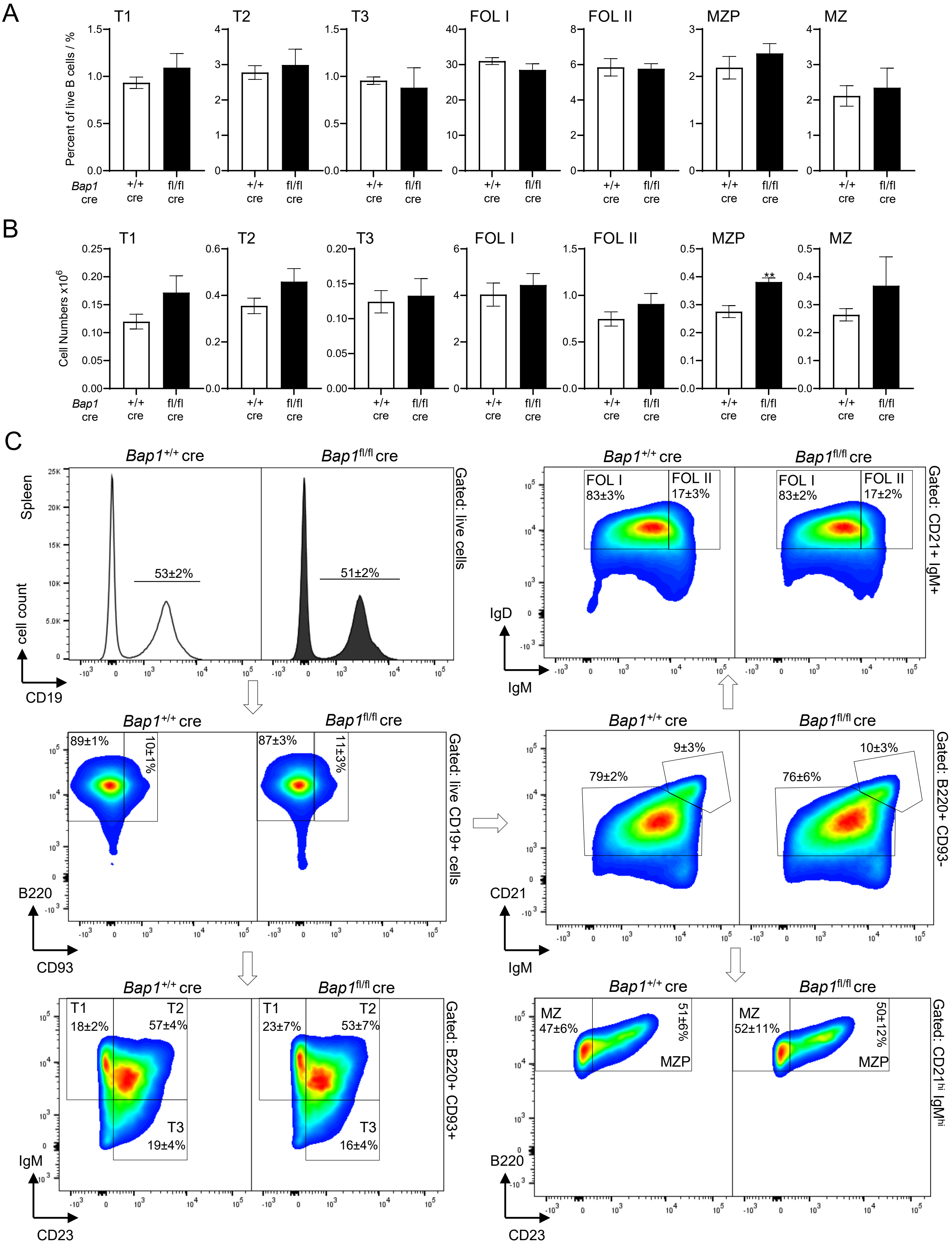
A
T1
T2
T3
FOL I
FOL II
MZP
MZ
Percent of live B cells / %
Bap1
+/+ cre
fl/fl cre
B
Cell Numbers x10^6
**
C
Bap1+/+ cre
Bap1fl/fl cre
Spleen
cell count
CD19
53±2%
51±2%
Gated: live cells
89±1%
10±1%
87±3%
11±3%
B220
CD93
Gated: live CD19+ cells
T1
18±2%
T2
57±4%
T3
19±4%
T1
23±7%
T2
53±7%
T3
16±4%
IgM
CD23
Gated: B220+ CD93+
FOL I
83±3%
FOL II
17±3%
FOL I
83±2%
FOL II
17±2%
IgD
IgM
Gated: CD21+ IgM+
9±3%
79±2%
10±3%
76±6%
CD21
IgM
Gated: B220+ CD93-
MZ
47±6%
51±6%
MZP
MZ
52±11%
50±12%
MZP
B220
CD23
Gated: CD21hi IgMhi

Figure S3. Confirmation of Cre-mediated *Bap1*-gene inactivation in stimulated *Bap1*$^{fl/fl}$ Cγ1-cre B cells. (A-B) Genomic PCR analyses of splenic B cells of *Bap1*$^{fl/fl}$ Cγ1-cre and control *Bap1*$^{+/+}$ Cγ1-cre genotypes, with and without *in vitro* stimulation with LPS + IL-4 or anti-CD40 + IL-4. Tail-clip samples from the same mice are included as additional negative controls. **(A)** Loss of *Bap1*-floxed allele and **(B)** gain of *Bap1*-null allele in the stimulated *Bap1*$^{fl/fl}$ Cγ1-cre B cells but not in control samples. PCR primers were *Bap1*_Fw TGGGGATGTCTGGGGTAAAG with **(A)** *Bap1*_Rv TGGTGGCAAATGAGACCTTG and **(B)** *Bap1*_ex13_Rv2 AGTGCCATCCTACTCAGCAAA. **(C-D)** Loss of expression of the *Bap1*-floxed exons 6-12 in the RNA-seq data from primary GC B cells of *Bap1*$^{fl/fl}$ Cγ1-cre genotype relative to control *Bap1*$^{+/+}$ Cγ1-cre genotype. **(C)** Dot plot showing the number of sequencing reads mapped to *Bap1* exons 6-12 in the RNA-seq data from GC B cells of *Bap1*$^{fl/fl}$ Cγ1-cre and control *Bap1*$^{+/+}$ Cγ1-cre genotypes. Statistical analysis used Student's *t*-test; *** $p < 0.001$. **(D)** Genomic snapshots of the RNA-seq tracks of *Bap1* floxed exons 6-12 from GC B cells of *Bap1*$^{fl/fl}$ Cγ1-cre and control *Bap1*$^{+/+}$ Cγ1-cre genotypes. The maximum data range (y-axis scale) of the tracks is 12.

Figure S3

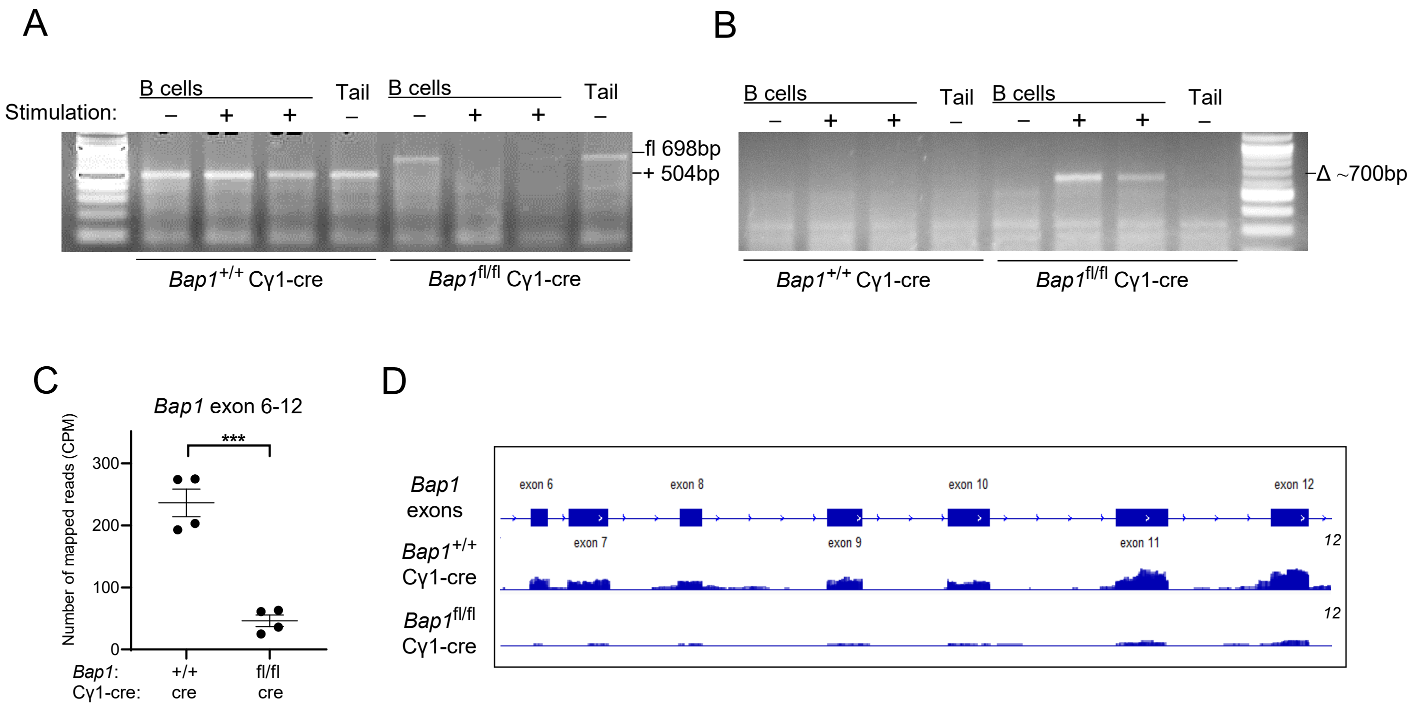

A
B cells
Tail
B cells
Tail
Stimulation:
–
+
+
–
–
+
+
–
fl 698bp
+ 504bp
Bap1+/+ Cγ1-cre
Bap1fl/fl Cγ1-cre
B
B cells
Tail
B cells
Tail
Δ ~700bp
Bap1+/+ Cγ1-cre
Bap1fl/fl Cγ1-cre
C
Bap1 exon 6-12
***
Number of mapped reads (CPM)
300
200
100
0
Bap1:
Cγ1-cre:
+/+
cre
fl/fl
cre
D
Bap1 exons
exon 6
exon 7
exon 8
exon 9
exon 10
exon 11
exon 12
Bap1+/+ Cγ1-cre
12
Bap1fl/fl Cγ1-cre
12

Figure S4. Further analyses of plasma cells in *Bap1*$^{fl/fl}$ Cγ1-*Cre* mice. Mice of *Bap1*$^{fl/fl}$ Cγ1-*Cre* and control *Bap1*$^{+/+}$ Cγ1-*Cre* genotypes, both naïve and at day 11 post-primary SRBC-immunization, were analyzed by flow cytometry. **(A)** Absolute numbers of plasmablasts, early plasma cells, and late plasma cells in mouse bone marrow (top panel) and spleen (bottom panel), comparing between the *Bap1* genotypes and immunization conditions. **(B)** Representative flow cytometry plots analyzing bone marrow plasma cells, gated as live Lin$^{–}$ CD138^{+} TACI $^{+}$, and showing the staining for the CD19^{+}B220^{+} plasmablast subpopulation; average percentage of cells in each gate relative to the parent gate for all the mice in each group is indicated as mean ± SD. **(C)** Analyses of bone marrow plasma cells for Ki-67 marker of cell proliferation, including quantification of the percentage of Ki-67+ plasma cells (left panel), and representative flow cytometry histogram gated on plasma cells and showing Ki-67 staining (right panel). All bars represent means ± SEM; statistical analyses used ANOVA with Sidak's post-hoc test to compare between the *Bap1*$^{fl/fl}$ Cγ1-*Cre* and *Bap1*$^{+/+}$ Cγ1-Cre genotypes and immunization conditions; * p<0.05, ** p<0.01, *** p<0.001. Plasma cells were gated as live CD138^{+} TACI^{+} cells, negative for the lineage markers CD11b, TER119, CD4, CD8, and NK1.1, and classified into CD19^{+}B220^{+} plasmablasts, CD19^{+}B220$^{–}$ early plasma cells, and CD19$^{–}$B220$^{–}$ late plasma cells. Intracellular staining for BLIMP1 transcription factor was used to validate plasma cell gating (data not shown). Furthermore, alternative staining for plasmablasts as B220^{hi} Ki67^{+}, early plasma cells as BLIMP1^{+} B220^{lo} Ki67$^{–}$, and late plasma cells as BLIMP1^{+} B220$^{–}$ Ki67$^{–}$ gave similar results and conclusions (data not shown).

Figure S4

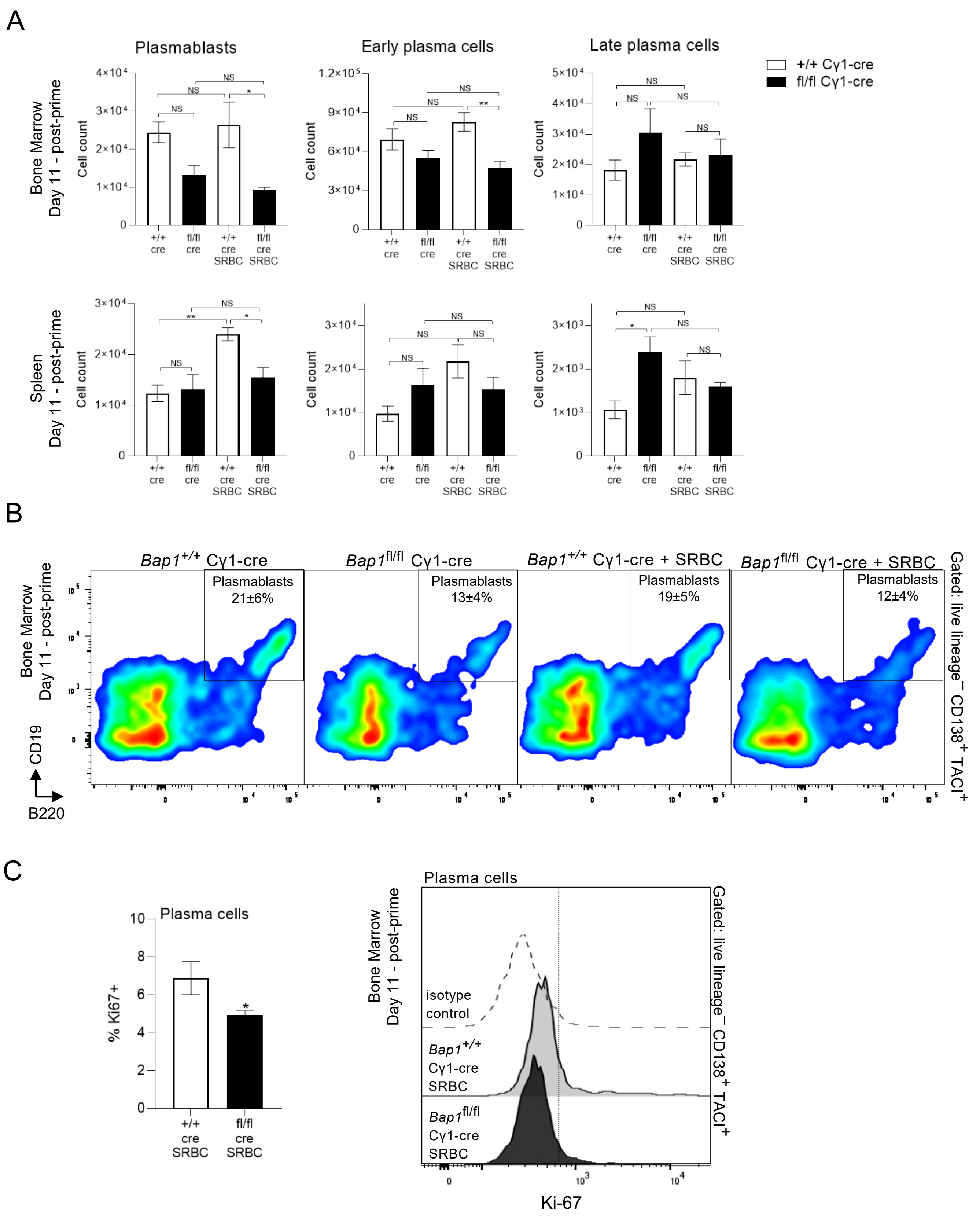

A
Plasmablasts
Early plasma cells
Late plasma cells
+/+ Cγ1-cre
fl/fl Cγ1-cre
Bone Marrow
Day 11 - post-prime
Spleen
Day 11 - post-prime
Cell count
NS
*
**
+/+ cre
fl/fl cre
+/+ cre SRBC
fl/fl cre SRBC
B
Bap1+/+ Cγ1-cre
Bap1fl/fl Cγ1-cre
Bap1+/+ Cγ1-cre + SRBC
Bap1fl/fl Cγ1-cre + SRBC
Plasmablasts 21±6%
Plasmablasts 13±4%
Plasmablasts 19±5%
Plasmablasts 12±4%
Gated: live lineage− CD138+ TACI+
CD19
B220
C
Plasma cells
% Ki67+
isotype control
Bap1+/+ Cγ1-cre SRBC
Bap1fl/fl Cγ1-cre SRBC
Ki-67

Figure S5. Analyses of germinal centre (GC) B cells, memory B cells, and plasma cells in *Bap1*$^{fl/fl}$ Cγ1-*Cre* mice post-boost immunization. Mice of *Bap1*$^{fl/fl}$ Cγ1-*Cre* and control *Bap1*$^{+/+}$ Cγ1-*Cre* genotypes, both naïve and at day 7 post-boost SRBC-immunization, were analyzed by flow cytometry. **(A)** Absolute numbers of GC B cells per mouse spleen, **(B)** ratio of dark zone (DZ) to light zone (LZ) GC B cells, **(C)** absolute numbers of IgG1^{+} GC B cells per mouse spleen, and **(D)** absolute numbers of IgG1^{+} memory B cells per mouse spleen, comparing between the *Bap1* genotypes and immunization conditions. **(E)** Representative flow cytometry plots analyzing splenic GC B cells; average percentage of cells in each gate relative to the parent gate for all the mice in each group is indicated as mean ± SD. **(F-G)** Absolute numbers of total plasma cells and IgG1^{+} plasma cells in the bone marrow and spleen, comparing between the *Bap1* genotypes and immunization conditions. **(H)** Representative flow cytometry analyses of plasma cells in the spleen of *Bap1*$^{fl/fl}$ Cγ1-*Cre* and control *Bap1*$^{+/+}$ Cγ1-*Cre* mice, both naïve and at day 7 post-boost SRBC-immunization; average percentage of cells in each gate relative to the parent gate for all the mice in each group is indicated as mean ± SD. Bars represent means ± SEM; statistical analyses used ANOVA with Sidak's post-hoc test to compare between the *Bap1*$^{fl/fl}$ Cγ1-*Cre* and *Bap1*$^{+/+}$ Cγ1-Cre genotypes and immunization conditions; * p<0.05, ** p<0.01, *** p<0.001. GC B cells were gated as live B220^{+}GL7^{+}CD95^{+} cells and divided into CXCR4^{+}CD86^{-} dark zone (DZ) and CXCR4^{-}CD86^{+} light zone (LZ) cell. Plasma cells were gated as live CD138^{+}TACI^{+} cells, negative for the lineage markers CD11b, TER119, CD4, CD8, and NK1.1.

Figure S5

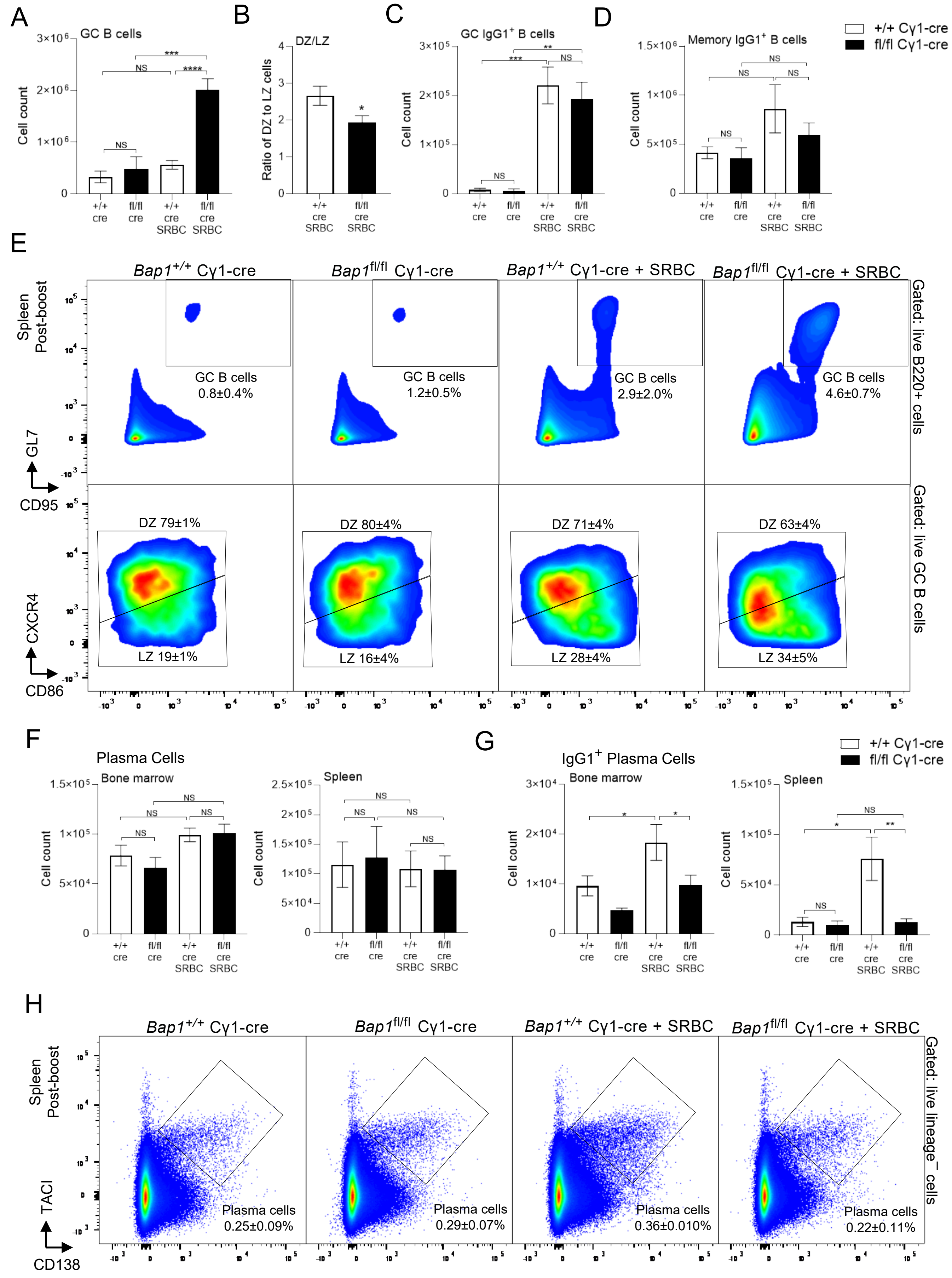

A
GC B cells
Cell count
NS
***
****
B
DZ/LZ
Ratio of DZ to LZ cells
*
C
GC IgG1+ B cells
D
Memory IgG1+ B cells
+/+ Cγ1-cre
fl/fl Cγ1-cre
+/+ cre
fl/fl cre
+/+ cre SRBC
fl/fl cre SRBC
E
Bap1+/+ Cγ1-cre
Bap1fl/fl Cγ1-cre
Bap1+/+ Cγ1-cre + SRBC
Bap1fl/fl Cγ1-cre + SRBC
Spleen Post-boost
Gated: live B220+ cells
GC B cells 0.8±0.4%
GC B cells 1.2±0.5%
GC B cells 2.9±2.0%
GC B cells 4.6±0.7%
GL7
CD95
Gated: live GC B cells
DZ 79±1%
LZ 19±1%
DZ 80±4%
LZ 16±4%
DZ 71±4%
LZ 28±4%
DZ 63±4%
LZ 34±5%
CXCR4
CD86
F
Plasma Cells
Bone marrow
Spleen
G
IgG1+ Plasma Cells
H
Gated: live lineage⁻ cells
Plasma cells 0.25±0.09%
Plasma cells 0.29±0.07%
Plasma cells 0.36±0.010%
Plasma cells 0.22±0.11%
TACI
CD138

Figure S6. Partial *Bap1*-deletion does not impair immunoglobulin class switching to IgA isotype in primary B cells. Primary splenic B cells from *Bap1*$^{fl/fl}$ Cγ1-*Cre* and control *Bap1*$^{+/+}$ Cγ1-Cre mice were stimulated in culture to induce class switching to IgA. **(A)** Analyses of the B cell cultures on day 4 of stimulation for class-switching to IgA and for cell proliferation using the CFSE-dilution method demonstrated no differences between the *Bap1*-genotypes. **(B)** Analyses of the B cell cultures for class-switching to IgA quantified per cell division demonstrated no differences between the *Bap1*-genotypes. **(C-D)** Genomic PCR analyses of the *Bap1*$^{fl/fl}$ Cγ1-cre and control *Bap1*$^{+/+}$ Cγ1-cre B cell cultures, either unstimulated on day 0 or on days 3-4 of stimulation. Gain of the *Bap1*-null allele **(C)**, but without the loss of the *Bap1*^{fl} allele **(D)** in stimulated *Bap1*$^{fl/fl}$ Cγ1-cre B cells is demonstrated, indicating that Cγ1-cre is only partially effective. PCR primers were *Bap1*_Fw TGGGGATGTCTGGGGTAAAG with **(C)** *Bap1*_ex13_Rv2 AGTGCCATCCTACTCAGCAAA or **(D)** *Bap1*_Rv TGGTGGCAAATGAGACCTTG.

Figure S6

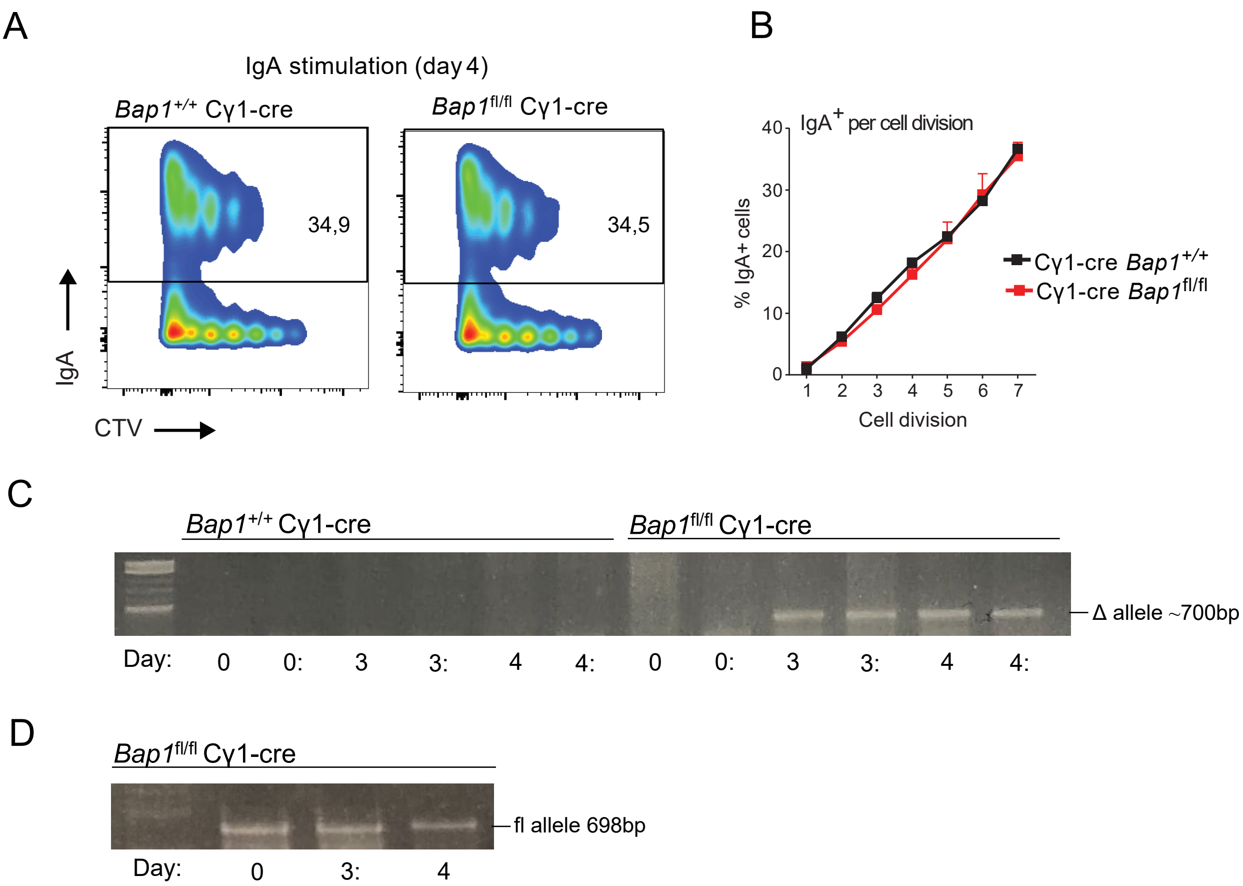
A
IgA stimulation (day 4)
Bap1+/+ Cγ1-cre
Bap1fl/fl Cγ1-cre
34,9
34,5
IgA
CTV
B
IgA+ per cell division
% IgA+ cells
40
30
20
10
0
1 2 3 4 5 6 7
Cell division
Cγ1-cre Bap1+/+
Cγ1-cre Bap1fl/fl
C
Bap1+/+ Cγ1-cre
Bap1fl/fl Cγ1-cre
Δ allele ~700bp
Day: 0 0: 3 3: 4 4: 0 0: 3 3: 4 4:
D
Bap1fl/fl Cγ1-cre
fl allele 698bp
Day: 0 3: 4

Figure S7. Analyses of the viability of *Bap1*-deficient B cells. (A-D) Viability of primary B cells from the spleen and bone marrow of the mice of *Bap1*$^{fl/fl}$ Cγ1-*Cre* and control *Bap1*$^{+/+}$ Cγ1-*Cre* genotypes, either naïve or at day 11 post-primary SRBC-immunization, analyzed by flow cytometry based on fixable viability dye exclusion. **(A)** Splenic germinal centre (GC) B cells, dark zone GC B cells, light zone GC B cells, and IgG1^{+} GC B cells, **(B)** splenic IgG1^{+} memory B cells, and **(C-D)** total plasma cells and IgG1^{+} plasma cells in the bone marrow and spleen show no differences in cell viability between the *Bap1*-genotypes. GC B cells were gated as live B220^{+}GL7^{+}CD95^{+} cells and divided into CXCR4^{+}CD86^{-} dark zone (DZ) and CXCR4^{-}CD86^{+} light zone (LZ) cell. Plasma cells were gated as live CD138^{+}TACI^{+} cells, negative for the lineage markers CD11b, TER119, CD4, CD8, and NK1.1. Bars represent means ± SEM; statistical analyses used ANOVA with Sidak's post-hoc test to compare *Bap1*$^{fl/fl}$ Cγ1-*Cre* and *Bap1*$^{+/+}$ Cγ1-Cre groups; no significant differences in B cell viability based on the *Bap1*-genotype were detected. **(E)** Viability of primary splenic B cells of *Bap1*$^{fl/fl}$ Cγ1-*Cre* and control *Bap1*$^{+/+}$ Cγ1-*Cre* genotypes at day 5 of *in vitro* stimulation with anti-CD40 (2 μg/ml) and IL-4 (5 ng/ml), or with LPS (1 μg/mL) and IL-4 (5 ng/ml), analyzed by flow cytometry based on fixable viability dye exclusion. Bars represent means ± SEM; statistical analyses used used Student's *t*-test; ** p<0.01, *** p<0.001.

Figure S7

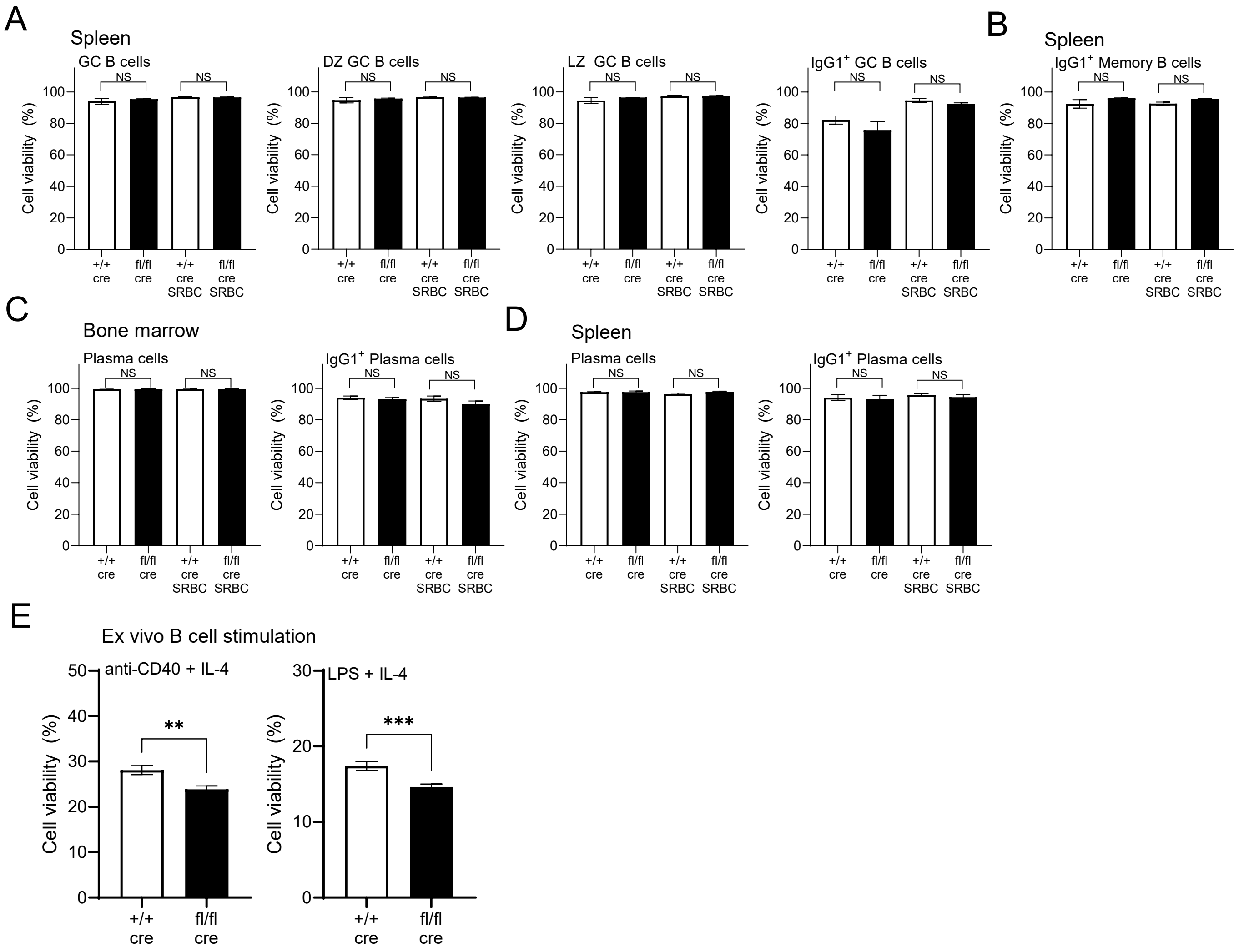
A
Spleen
GC B cells
DZ GC B cells
LZ GC B cells
IgG1+ GC B cells
B
Spleen
IgG1+ Memory B cells
C
Bone marrow
Plasma cells
IgG1+ Plasma cells
D
Spleen
Plasma cells
IgG1+ Plasma cells
NS
Cell viability (%)
+/+ cre
fl/fl cre
+/+ cre SRBC
fl/fl cre SRBC
E
Ex vivo B cell stimulation
anti-CD40 + IL-4
LPS + IL-4
**
***

Figure S8. CRISPR/Cas9-mediated *Bap1*-deletion in CH12F3 B cells, and the impaired proliferation of *Bap1*-deficient CH12F3 cell clones. (A) *Bap1* gene targeting strategy: *gRNA1* was designed to target exon 4, and *gRNA2* and *gRNA3* were designed to target exon 5 of *Bap1* gene, encoding the BAP1 catalytic domain (mm9 genome assembly). gRNAs were used in pairs to improve the efficiency of PCR screening for the deletions. The diagram shows the gRNA-targeted fragments of exons 4 and 5, as well as the corresponding amino acids of BAP1 protein. Arrows indicate the predicted sites of Cas9-mediated double strand breaks (DSB), and the key catalytic residue of BAP1 protein Cys91 is indicated in red. **(B)** Western blot validating the loss of BAP1 protein expression in *Bap1*$^{\Delta/\Delta}$ CH12F3 clones 1-5, using β-Actin as the loading control. The CH12F3 clones with successful *Bap1*-deletions were targeted with *gRNA2* and *gRNA3*. **(C-E)** Analyses of the effects of BAP1 loss of CH12F3 cell proliferation. **(C)** AlamarBlue assay demonstrates reduced proliferation of *Bap1*$^{\Delta/\Delta}$ CH12F3 cells; data is averaged from clones 1-5. **(D-E)** Cell proliferation analyses using a flow cytometry based CFSE-dilution assay, with the CH12F3 cells pre-loaded with CFSE and maintained in culture overnight. **(D)** Quantification of the CFSE mean fluorescence intensity (MFI) of *Bap1*$^{\Delta/\Delta}$ CH12F3 clones 1-5 relative to control wild type CH12F3 cells, demonstrating the reduced proliferation of *Bap1*$^{\Delta/\Delta}$ CH12F3 cells. **(E)** Representative CFSE dilution histograms, gated on live cells and demonstrating reduced proliferation of *Bap1*$^{\Delta/\Delta}$ relative to control wild type CH12F3 cells. **(F)** Viability of CH12F3 cells, analyzed by flow cytometry based on fixable viability dye exclusion, comparing *Bap1*$^{\Delta/\Delta}$ clones 1-5 to control wild type cells, using either unstimulated cells or cells at day 3 of stimulation with TGF-β (1 ng/ml), IL-4 (10 ng/ml), and anti–mouse CD40 (1μg/ml). **(C-D, F)** Bars represent mean ± SEM; statistical analyses used Student's *t*-test; * $p<0.05$, *** $p<0.001$, ns – not significant.

Figure S8

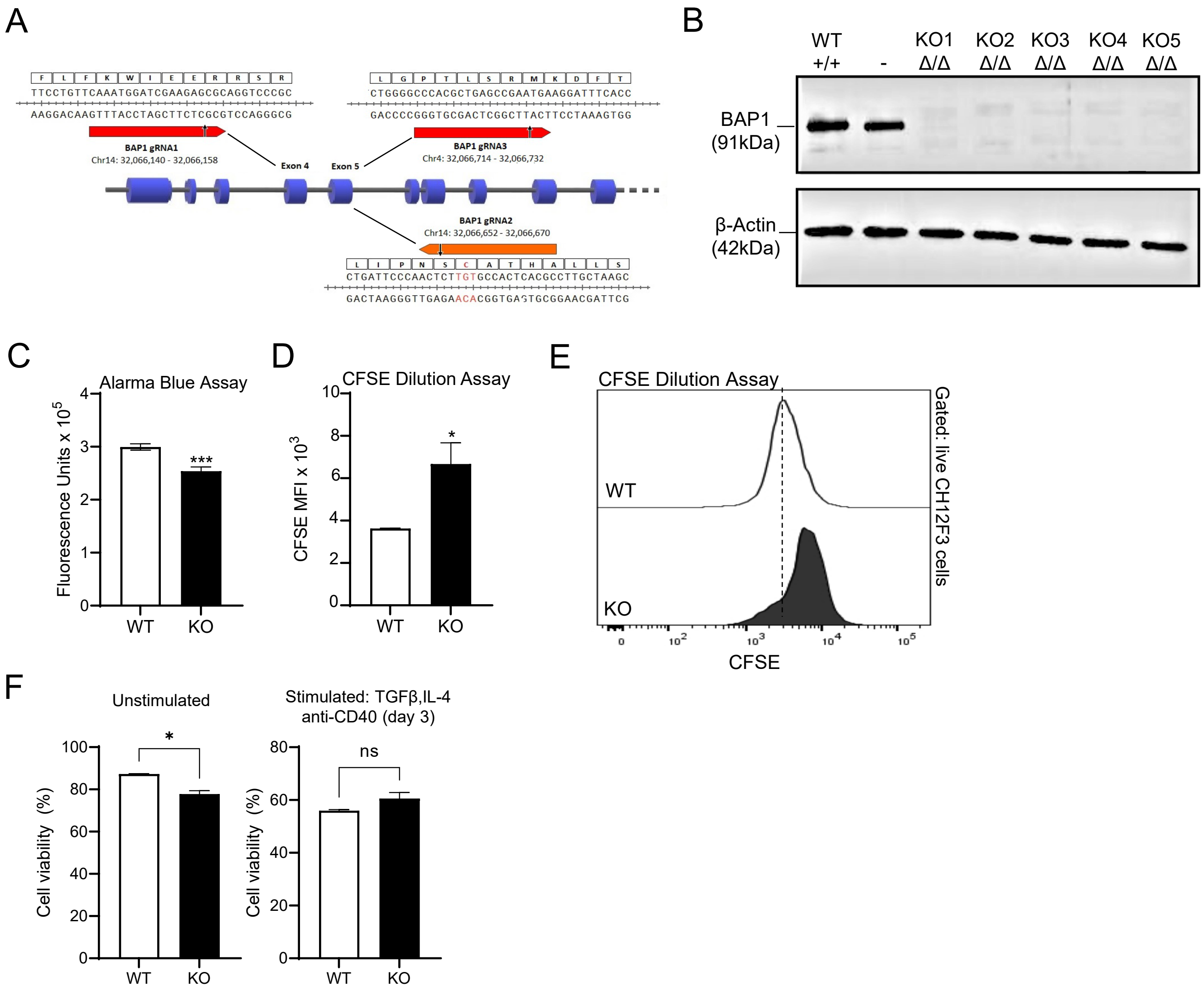

A
F L F K W I E E R R S R
TTCCTGTTCAAATGGATCGAAGAGCGCAGGTCCCGC
AAGGACAAGTTTACCTAGCTTCTCGCGTCCAGGGCG
BAP1 gRNA1
Chr14: 32,066,140 - 32,066,158
Exon 4
Exon 5
L G P T L S R M K D F T
CTGGGGCCCACGCTGAGCCGAATGAAGGATTTCACC
GACCCCGGGTGCGACTCGGCTTACTTCCTAAAGTGG
BAP1 gRNA3
Chr4: 32,066,714 - 32,066,732
BAP1 gRNA2
Chr14: 32,066,652 - 32,066,670
L I P N S C A T H A L L S
CTGATTCCCAACTCTTGTGCCACTCACGCCTTGCTAAGC
GACTAAGGGTTGAGAACACGGTGAGTGCGGAACGATTCG
B
WT +/+
-
KO1 Δ/Δ
KO2 Δ/Δ
KO3 Δ/Δ
KO4 Δ/Δ
KO5 Δ/Δ
BAP1 (91kDa)
β-Actin (42kDa)
C
Alarma Blue Assay
Fluorescence Units x 10^5
0 1 2 3 4
***
WT KO
D
CFSE Dilution Assay
CFSE MFI x 10^3
0 2 4 6 8 10
*
WT KO
E
CFSE Dilution Assay
Gated: live CH12F3 cells
WT
KO
0 10^2 10^3 10^4 10^5
CFSE
F
Unstimulated
Cell viability (%)
0 20 40 60 80 100
*
WT KO
Stimulated: TGFβ,IL-4
anti-CD40 (day 3)
Cell viability (%)
0 20 40 60 80
ns
WT KO

Figure S9. ChIP-seq analyses of the BAP1 genomic binding sites in CH12F3 cells in consolidation with other relevant public ChIP-seq datasets. (A-B) Heatmaps showing the tag densities of 1,499 BAP1 binding sites identified in our ChIP-seq analyses of CH12F3 cells, including from left to right wild type (WT) cells analyzed with anti-BAP1 antibody, cells stably expressing FLAGx3-tagged BAP1 analyzed with anti-FLAG antibody, and WT cells stimulated with TGFβ, IL-4, and anti-CD40 for 72 hours and analyzed with anti-BAP1 antibody. **(A)** The BAP1 ChIP-seq datasets from CH12F3 cells are consolidated with the available public BAP1 ChIP-seq datasets, including from Ba/F3 pro-B cells (1), ES cells (3), and bone marrow derived macrophages (2), to analyze for BAP1 binding site colocalization across different cell types. **(B)** The BAP1 ChIP-seq datasets from CH12F3 cells are consolidated with the public ChIP-seq datasets for BAP1 associated transcriptional regulators, including HCF1 and OGT in bone marrow derived macrophages (2) and ASXL1 in bone marrow hematopoietic stem and progenitor cells (HSPCs, cKit^{+}) (4), as well as ChIP-seq datasets for other regulators of histone H2AK119ub, including polycomb proteins RING1B, CBX7, EZH2 and deubiquitinase USP16 from quiescent CD43^{-} resting splenic B cells (5) and polycomb protein YY1 from follicular splenic B cells (CD19^{+}AA4^{-}CD21^{lo}CD23^{hi}) (6). All public ChIP-seq datasets are from mouse, downloaded and re-analyzed using our pipelines.

Figure S9

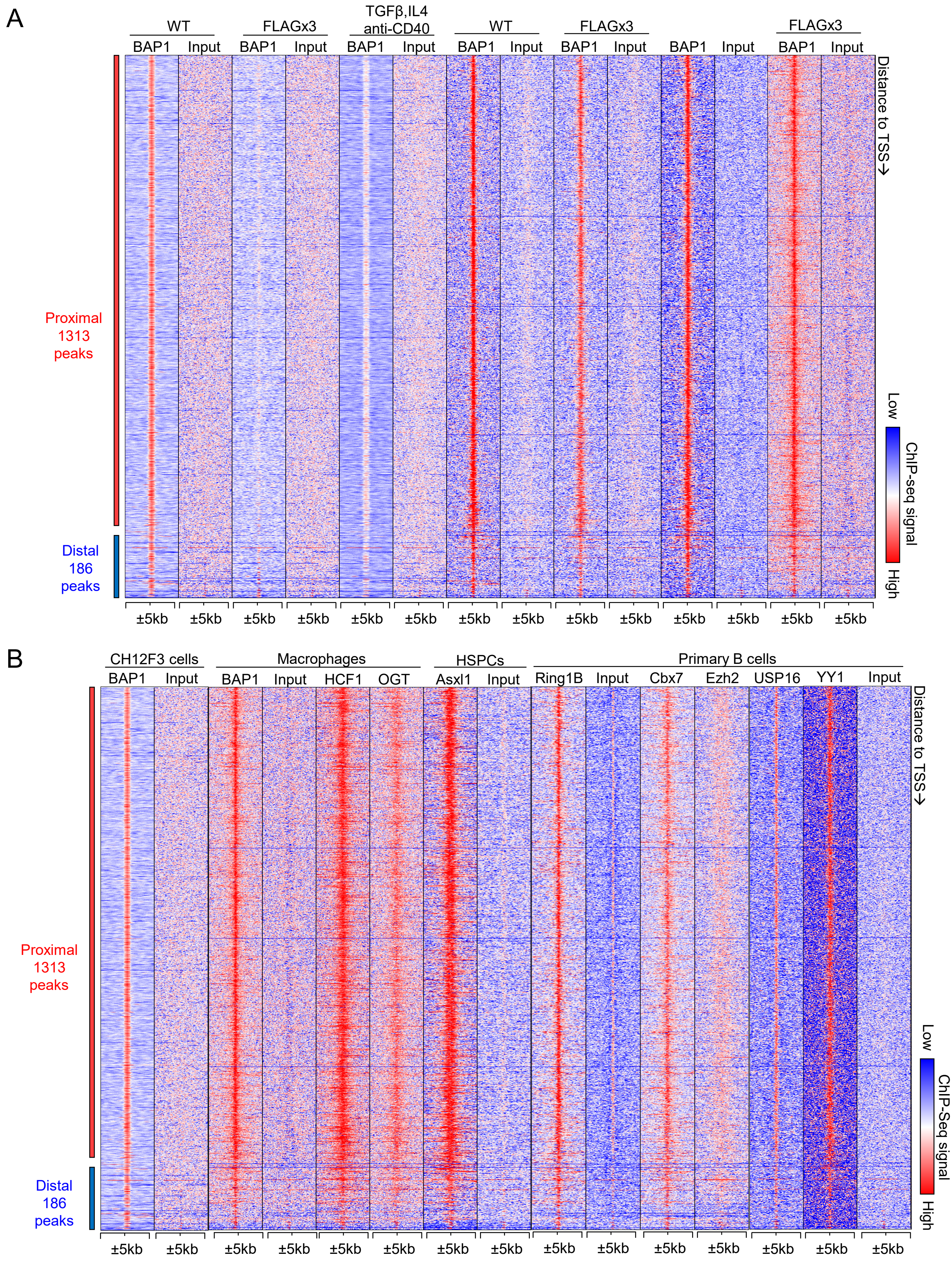
A
CH12F3 cells
WT
FLAGx3
TGFβ,IL4
anti-CD40
Ba/F3 cells
ES cells
Macrophages
BAP1
Input
Distance to TSS→
Proximal
1313
peaks
Distal
186
peaks
Low
ChIP-seq signal
High
±5kb
B
CH12F3 cells
Macrophages
HSPCs
Primary B cells
BAP1
Input
HCF1
OGT
Asxl1
Ring1B
Cbx7
Ezh2
USP16
YY1
ChIP-Seq signal

Figure S10. Gating strategy used for the FACS-sort of GC B cells for RNA-seq analyses. GC B cells were FACS-sorted from total splenocytes of *Bap1*$^{fl/fl}$ *Cγ1*-cre and control *Bap1*$^{+/+}$ *Cγ1*-cre mice at day 11 after the primary intravenous immunization with SRBC. Representative flow cytometry plots show the gating for GC B cell population as live B220^{+}GL7^{+}CD95^{+} cells. Percentages of cells within each gate relative to the parent gate for each mouse genotype are presented as mean ± S.D.

Figure S10

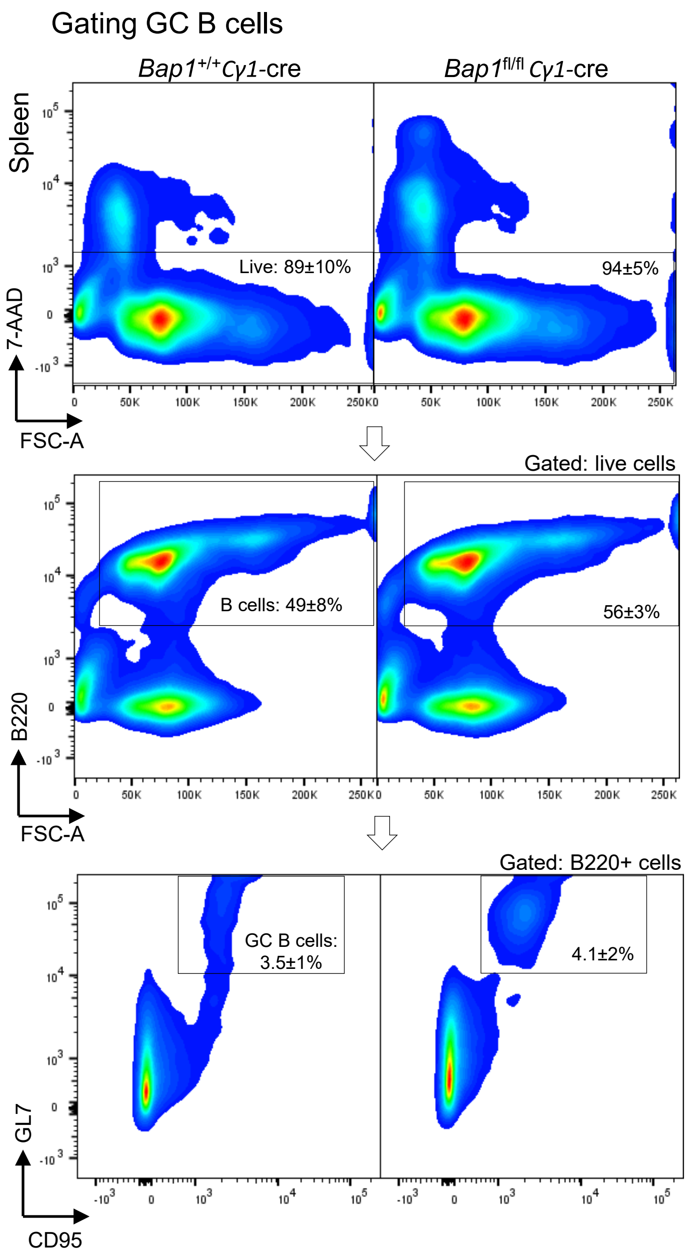
Gating GC B cells
Bap1+/+Cγ1-cre
Bap1fl/fl Cγ1-cre
Spleen
Live: 89±10%
94±5%
7-AAD
FSC-A
Gated: live cells
B cells: 49±8%
56±3%
B220
FSC-A
Gated: B220+ cells
GC B cells: 3.5±1%
4.1±2%
GL7
CD95

REFERENCES

1. Lin YH, Liang Y, Wang H, Tung LT, Forster M, Subramani PG, et al. Regulation of B Lymphocyte Development by Histone H2A Deubiquitinase BAP1. Front Immunol. 2021;12:626418.
2. Dey A, Seshasayee D, Noubade R, French DM, Liu J, Chaurushiya MS, et al. Loss of the tumor suppressor BAP1 causes myeloid transformation. Science. 2012;337(6101):1541-6.
3. Kweon SM, Chen Y, Moon E, Kvederaviciute K, Klimasauskas S, Feldman DE. An Adversarial DNA N(6)-Methyladenine-Sensor Network Preserves Polycomb Silencing. Mol Cell. 2019;74(6):1138-47 e6.
4. Micol JB, Pastore A, Inoue D, Duployez N, Kim E, Lee SC, et al. ASXL2 is essential for haematopoiesis and acts as a haploinsufficient tumour suppressor in leukemia. Nat Commun. 2017;8:15429.
5. Frangini A, Sjoberg M, Roman-Trufero M, Dharmalingam G, Haberle V, Bartke T, et al. The aurora B kinase and the polycomb protein ring1B combine to regulate active promoters in quiescent lymphocytes. Mol Cell. 2013;51(5):647-61.
6. Kleiman E, Jia H, Loguercio S, Su AI, Feeney AJ. YY1 plays an essential role at all stages of B-cell differentiation. Proc Natl Acad Sci U S A. 2016;113(27):E3911-20.
7. Delgado-Benito V, Rosen DB, Wang Q, Gazumyan A, Pai JA, Oliveira TY, et al. The Chromatin Reader ZMYND8 Regulates Igh Enhancers to Promote Immunoglobulin Class Switch Recombination. Mol Cell. 2018;72(4):636-49 e8.
8. Yoshida H, Lareau CA, Ramirez RN, Rose SA, Maier B, Wroblewska A, et al. The cis-Regulatory Atlas of the Mouse Immune System. Cell. 2019;176(4):897-912 e20.
9. Wang H, Langlais D, Nijnik A. Histone H2A deubiquitinases in the transcriptional programs of development and hematopoiesis: a consolidated analysis. Int J Biochem Cell Biol. 2023;157:106384.
10. Allman D, Pillai S. Peripheral B cell subsets. Curr Opin Immunol. 2008;20(2):149-57.

Graphical Abstract

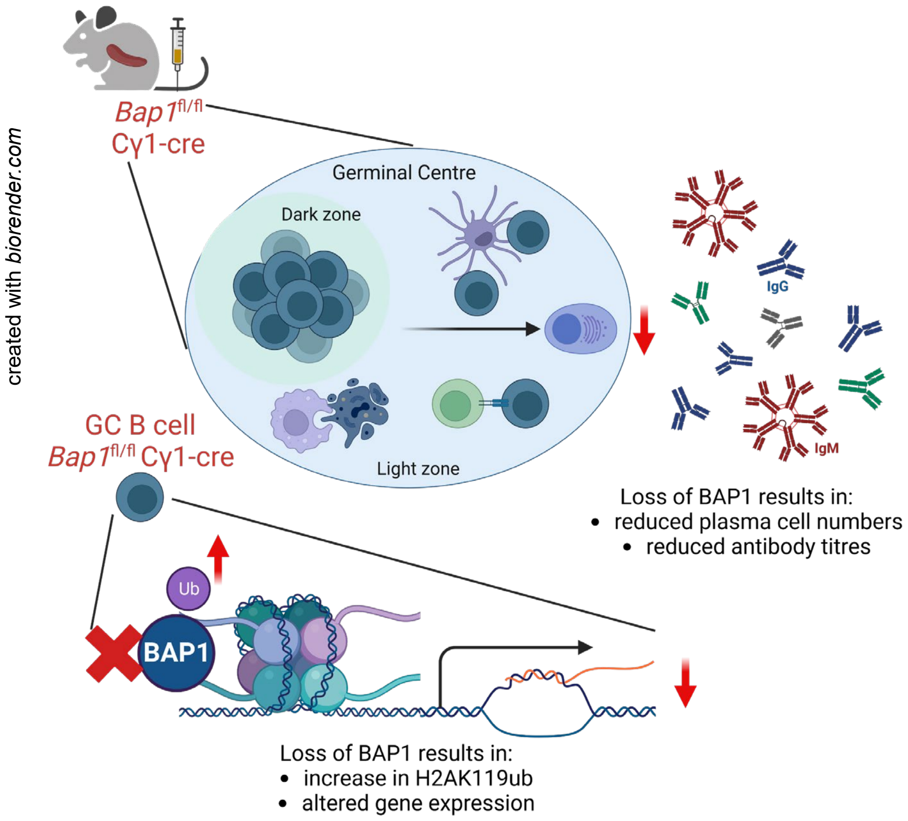

Supplement: Supplementary file 1 [file DataSheet_1.pdf]
